# The Hypolipidemic Effect of Hawthorn Leaf Flavonoids through Modulating Lipid Metabolism and Gut Microbiota in Hyperlipidemic Rats

**DOI:** 10.1155/2022/3033311

**Published:** 2022-11-15

**Authors:** Huiming Hu, Jiajun Weng, Can Cui, Fangrui Tang, Mengdan Yu, Yujie Zhou, Feng Shao, Yanchen Zhu

**Affiliations:** ^1^Science and Technology College, Jiangxi University of Chinese Medicine, Nanchang 330004, Jiangxi, China; ^2^The Key Laboratory of Health Pharmacodynamics and Safety Evaluation of Jiangxi, Nanchang Medical College, Nanchang 330052, Jiangxi, China; ^3^Peking University Traditional Chinese Medicine Clinical Medical School (Xiyuan), Beijing 100191, China; ^4^Key Laboratory of Modern Preparation of Traditional Chinese Medicine, Ministry of Education, Jiangxi University of Chinese Medicine, Nanchang 330004, Jiangxi, China; ^5^College of Pharmacy, Jiangxi University of Chinese Medicine, Nanchang 330004, Jiangxi, China; ^6^College of Computer Science, Jiangxi University of Chinese Medicine, Nanchang 330004, Jiangxi, China

## Abstract

**Objective:**

The purpose of this study was to explore the potential mechanisms of the lipid-regulating effects and the effect on modulating the gut microbiota of hawthorn leaf flavonoids (HLF) in the high-fat diet-induced hyperlipidemic rats.

**Methods:**

The hypolipidemic effect of HLF was investigated in the high-fat diet-induced hyperlipidemic rats. The action targets of HLF in the treatment of hyperlipidemia were predicted by network pharmacology and KEGG enrichment bubble diagram, which were verified by the test of western blotting. Meanwhile, we used 16S rRNA sequencing to evaluate the effects of HLF on the microbes.

**Results:**

The results of animal experiments showed that HLF could reduce the body weight and regulate the levels of serum lipid in high-fat diet (HFD) rats. Meanwhile, for the related targets of cholesterol metabolism, HLF could significantly upregulate the expression of LDLR, NR1H3, and ABCG5/ABCG8; reduce the expression of PCSK9; and increase the level of CYP7A1 in the intestinal tissue, whereas cholesterol biosynthetic protein expressions including HMGCR and SCAP were lowered by HLF. In addition, HLF increased the activities of plasma SOD, CAT, and GSH-Px and decreased the levels of Casp 1, NLRP3, IL-1*β*, IL-18, and TNF-*α*, improving the degree of hepatocyte steatosis and inflammatory infiltration of rats. Notably, HLF significantly regulated the relative abundance of major bacteria such as *g_Lactobacillus*, *g_Anaerostipes*, *g_[Eubacterium]_hallii_group*, *g_Fusicatenibacter*, *g_Akkermansia*, and *g_Collinsella*. Synchronously, we found that HLF could regulate the disorder of plasma HEPC and TFR levels caused by HFD.

**Conclusion:**

This study demonstrates that HLF can regulate metabolic hyperlipidemia syndromes and modulate the relative abundance of major bacteria, which illustrated that it might be associated with the modulation of gut microbiota composition and metabolites.

## 1. Introduction

Hyperlipidemia, also known as dyslipidemia, refers to the increase of total cholesterol (TC), triglyceride (TG), and low-density lipoprotein cholesterol (LDL-C) and the decrease of high-density lipoprotein cholesterol (HDL-C) [1]. The pathological process of hyperlipidemia is closely related to the physiological and pathological processes of many tissues and cells, such as metabolism [2], inflammation [3], immunity, stress, and so on [4]. It is also an important risk factor for cardiovascular and metabolic diseases, such as atherosclerosis, fatty liver disease, obesity, hypertension, diabetes mellitus, coronary heart disease, and stroke [5, 6]. At present, the drug therapy for hyperlipidemia in clinic are mainly statins, fibrates, and niacins, among which statins are the first choice and are considered the cornerstone of preventing atherosclerotic and cardiovascular disease (ASCVD). In spite of the statins therapy-mediated positive effects on cardiovascular diseases, patient compliance is often poor due to their adverse effects [7]. It is worth mentioning that traditional Chinese medicine (TCM) has unique advantages that are low cost, effectiveness, and fewer side effects in the treatment of hyperlipidemia [8, 9].

Hawthorn (*Crataegi folium*) leaves are the dried leaves of *Crataegus pinnatifida* Bge. of the Rosaceae plant, which have the effects of lipid lowering [10], antiatherosclerosis [11], antiliver damage [12], anti-inflammation, and antioxidative stress [13]. A variety of hawthorn leaf preparations, such as Yixintong and Shanmei capsule, is clinically used to treat cardiovascular diseases such as hyperlipidemia, coronary heart disease, angina pectoris, and arrhythmia [14, 15]. At present, a great many types of chemical constituents have been extracted from hawthorn leaves, including flavonoids, flavane and its polymers, pentacyclic triterpenes, monoterpenes, sesquiterpenes, lignans, organic acids, volatile oil, and so on [10, 16, 17], among which flavonoids are considered being the main active ingredients of hawthorn leaves and important ingredients for the herb to exert its drug activity [18, 19]. Our previous study [20] found that hawthorn leaf flavonoids (HLF) can reduce the levels of blood lipid and improve the liver function in hyperlipidemic mice. Meanwhile, the protein expression profiles of HMGCR in the liver were downregulated by HLF. However, the specific mechanism by which HLF regulate lipid metabolism remains unclear.

Network pharmacology integrates multidisciplinary and multiomics databases, and systematically and integrally connects drugs and diseases through network construction tools, which can provide scientific, technological, and theoretical support for the study of the action mechanism of TCM [21–23]. For instance, baicalin could regulate the gene expression of SLC2A1, TNF, NFKB1, SREBF1, and CASP3 to ameliorate obesity and hyperlipidemia through a network pharmacology approach [24]. Furthermore, the cholesterol metabolism, fat digestion and absorption, and PPAR signaling pathways were identified as the potential mechanism of sea buckthorn flavonoids extract, among which isorhamnetin could activate the PPAR-*γ*/NR1H3/CYP7A1 pathway against HLP by network pharmacology analysis and experimental validation [25]. In this study, network pharmacology analysis was used to screen the active components of HLF to further predict the action targets of the active compounds. The mechanism of HLF in the treatment of hyperlipidemia was further analyzed by constructing a network diagram and KEGG enrichment bubble diagram.

Gut microbiota is a key environmental factor that regulates body metabolism [26]. A balanced gut microbiota can maintain lipid homeostasis through pathways such as regulating hepatic cholesterol metabolism, promoting muscle lipid oxidation and adipose tissue energy storage, and maintaining the integrity of the gut barrier [27, 28]. Imbalances in the gut microbiota can lead to proliferation of potential pathogenic bacteria, affect immune homeostasis, and induce the production of inflammatory cytokines and adipokines. It is closely associated with the development and progression of chronic diseases such as hyperlipidemia, obesity, diabetes, and atherosclerosis [29–31]. Meanwhile, gut mucosal barrier damage induced by a high-fat diet exacerbates this condition [32, 33]. It is demonstrated that the relative abundances of the beneficial genuses such as *Lactobacillus* and *Oscillibacter* were inhibited by the high-fat diet. Furthermore, *Auricularia auricula* and its polysaccharides could improve the intestinal microbial environment by enriching SCFA-producing bacteria to relieve liver damage and treat hyperlipidemia [34]. However, the microbiota-modulating effects of hawthorn leaf flavonoids on diet-induced hyperlipidemia rats have not been revealed yet.

Therefore, the hypolipidemic effect of HLF was investigated in high-fat diet-induced hyperlipidemic rats. The action targets of HLF in the treatment of hyperlipidemia were predicted by network pharmacology and KEGG enrichment bubble diagram, which were verified by molecular docking and the test of western blotting. Meanwhile, the composition and richness of gut microbiota were tested by 16S rRNA sequencing, and the correlations with HLF intervention were analyzed accordingly.

## 2. Materials and Methods

### 2.1. Materials and Drugs

Total cholesterol (TC) assay kit (lot no. 2020021001), triglyceride (TG) assay kit (lot no. 2020051901), high-density lipoprotein cholesterol (HDL-C) assay kit (lot no. 2020041001), and low-density lipoprotein cholesterol (LDL-C) assay kit (lot no. 2020061102) were offered by Epnkan Biological Technology CO., China. Rat sIgA ELISA kit (lot: 20210804. 60140R), rat cholesterol 7*α*-hydroxylase (CYP7A1) ELISA kit (lot no. 20210804. 60324R), rat caspase-1 (Casp1) ELISA kit (lot: 20210804. 60626R), rat NOD-like receptor protein 3 (NLRP3) ELISA kit (lot no. 20210804. 60629R), rat 3-hydroxy-3-methylglutaryl-coenzyme A reductase (HMGCR) ELISA kit (lot no. 20210804. 60597R), rat interleukin-1*β* (rat IL-1*β*) ELISA kit (lot no. 20210804. 60013R), rat interleukin-6 (rat IL-6) ELISA kit (lot no. 20210804. 60023R), rat interleukin-18 (rat IL-18) ELISA kit (lot no. 20210804. 60033R), rat tumor necrosis factor-alpha (TNF-*α*) ELISA kit (lot no. 20210804. 60080R), rat transferrin (TRF) ELISA kit (lot no. 20220425. 60655R), and rat hepcidin (HEPC) ELISA kit (lot no. 20220425. 60657R) were obtained from Beijing Rigorbio Science Development Co., Ltd., China. Total superoxide dismutase (SOD) assay kit (lot no. 20210802), catalase (CAT) assay kit (lot no. 20210805), and glutathione peroxidase (GSH-Px) assay kit (lot no. 20210803) were purchased from Nanjing Jiancheng Bioengineering Institute, China. Rabbit anti-SCAP antibody (bs-3862R), rabbit anti-HMGCR antibody (bsm-52822R), rabbit anti-LDL receptor antibody (bs-0705R), rabbit anti-PCSK9 antibody (bs-6060R), rabbit anti-NR1H3 antibody (bs-2342R), rabbit anti-ABCG5 (bs-5013R) antibody, and rabbit anti-ABCG8 (bs-10149R) antibody were purchased from Bioss, China. SREBF2 antibody (DF7601) was purchased from Internal Affinity Biosciences, USA. Anti-NLRP3 antibody (BA3677) was purchased from Boster Biological Technology Co., Ltd., USA. *β*-actin (4D3) polyclonal antibody (AP6007M) was obtained from Bioworld Technology, Inc, USA. Hawthorn leaf flavonoids (HLF) (no: ZLSC2020071501, content ≥90%) were purchased from Nanjing Zelang Biotechnology Co., Ltd., China.

### 2.2. Animals and Diets

Sprague–Dawley (SD) male rats (200 ± 20 g, 8 weeks, no. 1107272011005517) were purchased from Hunan SJA Laboratory Animal Co., Ltd (Hunan, China). Rats were kept at room temperature (24–26°C, 65% ± 10% humidity, and 12/12 h light/darkness cycle) with a commercial rat normal standard chow (Hunan SJA Laboratory Animal Co., Ltd., Hunan, China) and water ad libitum. After allowing 1 week for adaptation, all rats were assigned randomly into six groups (*n* = 8). The rats in the normal control group (NC) were fed with a standard basal diet, while rats in the other 5 groups were fed with the high-fat diet (52.6% regular diet, 20.0% sucrose, 15.0% lard, 1.2% cholesterol, 0.2% bile salts, 10% casein, 0.6% calcium hydrophosphate, and 0.4% mountain flour) to obtain the hyperlipidemic model [35]. After 2 weeks, rats in the NC and high-fat diet group (HFD) were intragastrically given 10 ml· kg^−1^ body weight (BW) of distilled water once a day. Rats in group 3∼5 were administered high-fat diet with HLF-L (100 mg/kg·day), HLF-M (200 mg/kg·day), and HLF-H (400 mg/kg day) [36], respectively. Rats in group 6 were administered high-fat diet with atorvastatin (AVT, 7 mg/kg day) [37]. All samples were dissolved in distilled water and intragastrically given at a dose of 10 ml·kg^−1^ once a day for 4 weeks.

### 2.3. Biochemical Analysis

Blood samples were taken from the orbit vein and subsequently centrifuged. The serum or plasma obtained was stored at −80°C until biochemical analysis. The serum lipid levels (TC, TG, LDL-C, and HDL-C) were determined by a Beckman Coulter AU480 Automatic Biochemical Analyzer (USA).

### 2.4. ELISA Analysis

The levels of rat IL-1*β*, IL-6, IL-18, TNF-*α*, and HMGCR in plasma and CYP7A1, NLRP3, sIgA, and Casp1 in the intestinal tissue were measured using commercial analysis kits by Thermo Multiskan MK3 Microplate Reader (Finland). The levels of rat SOD, CAT, GSH-Px, TRF, and HEPC in plasma were determined by a Beckman Coulter UniCel DxC 600 Synchron Automatic Biochemical Analyzer (USA).

### 2.5. Histological Analysis

To make paraffin sections, liver tissue specimens were fixed in 10% formalin, paraffin-embedded, and sectioned at 4 *μ*m. Hematoxylin and eosin (*H* & *E*) staining was according to the standard method by Dako CoverStainer.

### 2.6. Gut Microbiota Analysis

#### 2.6.1. Microbial DNA Extraction and 16S rRNA Sequencing of Feces

Before taking materials, clean the anus of rats with 75% ethanol cotton ball, take the natural excreted feces of rats with sterilized EP tubes, put them into liquid nitrogen immediately, and then transfer them to the refrigerator at −80°C for freezing storage. One week later, they were sent to Shanghai Majorbio Pharm Technology Co., Ltd, together with dry ice for sequencing. The integrity, purity, and concentration of DNA are detected by 1% agarose gel. Subsequently, primers with labels are designed and synthesized based on the v3–v4 region of 16S rRNA and amplified by PCR. All samples are conducted according to the formal experimental conditions. Each sample were replicated 3 times. The PCR products of the same sample were mixed and then detected by 2% agarose gel electrophoresis. The PCR products are recovered by gel extraction using AxyPrepDNA Gel Recovery Kit (AXYGEN company). Finally, referring to the preliminary quantitative results of electrophoresis, the PCR products are quantified with QuantiFluor™-ST blue fluorescence quantitative system (Promega company), and then the corresponding proportion is mixed according to the sequencing volume requirements of each sample. Use the library construction kit (TruSeqTM DNA Sample Prep Kit) of Illumina company to construct the library according to the standard process of MiSeq platform. After quantification and quality control, the obtained library is sequenced on the Illumina MiSeq PE300 sequencing platform (Shanghai Majorbio Pharm Technology Co., Ltd).

#### 2.6.2. Gut Microbiota Analysis

The PE reads obtained by MiSeq sequencing are first spliced according to the overlapping relationship to obtain the original sequence, which was filtered and quality controlled. Based on the Uparse software (v7.0.1090), we set the classification confidence to 70%. OTU clustering is performed for nonrepetitive sequences (excluding single sequences) according to 97% similarity and remove the chimera in this process. Then, compared with the Silva 128/16S bacteria database, annotate the optimized sequence to obtain taxonomic information and flatten the sample sequence according to the minimum number of sample sequences (*E*1: 34099) before the data analysis.

The alpha diversity index of the samples was evaluated by the Mothur software (version v.1.30.2), and the difference between the groups is analyzed. Use the *R* language tool to make the rank abundance curve explain the diversity, draw the pan/core species curve to judge whether the sample size is enough, evaluate the total species richness and the number of core species in the feces, and make the Venn chart and community bar chart to show the species' composition and similarity. Based on two distance algorithms (unweighted_unifrac and weighted_unifrac), principal coordinate analysis (PCoA), statistical analysis, and nonmetric multidimensional scaling analysis (NMDS) were carried out by using Qiime (version v. 1.9.1) as well as *R* language tools to calculate the distance between the samples and obtain the distance matrix. LEfSe software is used to analyze the difference of species' relative abundance between the groups. In this study, all-against-all (more-strict) comparison strategies and bacteria with linear discriminant analysis (LDA) score >3 and *p* < 0.05 were selected as differential bacteria.

In terms of alpha diversity, Student's test is used to test the difference of index values among the groups. In terms of beta diversity, ANOSIM is used to detect the difference of community composition among different groups. One-way ANOVA test and post hoc Tukey–Kramer test are used to compare the abundance of gut microbiota in each group. In LEfSe analysis, the nonparametric Kruskal–Wallis (KW) sum rank test is mainly used to detect the species' abundance differences among different groups and significantly different species are obtained. Then, Wilcoxon rank sum test is used to test the difference consistency of different species in different subgroups; finally, LDA (linear discriminant analysis) is used to estimate the impact of these different species on the difference between the groups.

### 2.7. Network Pharmacology Analysis

#### 2.7.1. Compound Collection and Target Prediction for HLF

The active ingredients of HLF are extracted from the Traditional Chinese Medicine Database and Analysis Platform (TCMSP) [38], the chemical database, and supplemented with references to the related literature. The acquired active substances are imported into the PubChem [39] for InChi and canonical smiles. The acquired InChi and canonical smiles are input into the Comparative Toxicogenomics Database (CTD) [40], STITCH [41] according to the method of compound similarity search, the species are defined as Homo sapiens, the potential targets of the active ingredients are predicted, and all target information is normalized using UniProt [42].

#### 2.7.2. Hyperlipidemia-Related Targets Collection and Screening

“Hyperlipidemia” has been retrieved from GeneCards [43], and the top 200 as potential targets for hyperlipidemia have been tested by top-down score. After all target names are corrected, merged, and removed duplicates and TCM targets are imported into the Jvenn [44] to draw a Venn diagram, and its intersection is taken to obtain 83 HLF antihyperlipidemia possible targets.

#### 2.7.3. PPI Network Construction

Proteins rarely function as a single substance, but as members in a dynamic network. The accumulation of evidence suggests that protein-protein interactions (PPI) are critical to many biological processes in living cells [45]. For clarity of the interaction relationship between HLF-related targets and hyperlipidemia targets, we submitted the 83 intersection targets to STRING [46] with the species set as “*Homo sapiens*”.

#### 2.7.4. Analysis of GO Function and KEGG Pathway Enrichment

Intersection targets are entered into the Metascape [47], setting the species to “*H. sapiens*,” min overlap = 3, *p* value cutoff = 0.01, min. enrichment = 1.5, and *p* < 0.01. Mainly analyze GO molecular functions (MF), GO components (CC), GO biological processes (BP), and KEGG pathway; save the data results; and import the Origin 2021 software to draw the bubble chart.

#### 2.7.5. Construction of the “Active Ingredient-Target-Biological Processes” Network Diagram

The −log  *p* top 25 biological processes and related components and targets are imported into the model using Cytoscape 3.8.2. Nodes are used to represent active ingredients and predicted targets, and nodes are connected to the edges to represent subordination. Network analysis is used to analyze network topological properties.

### 2.8. Western Blotting Assay

Total proteins were obtained from the rat hepatic tissue and intestinal tissue homogenates with RIPA buffer supplemented with phenylmethylsul-fonyl fluoride and protease inhibitor cocktail. Protein samples were separated on 10% separation gel and then transferred to polyvinylidene fluoride membranes. After blocking with 5% fetal bovine serum for 1 h, we then incubated separately with primary rabbit polyclonal antibodies against SCAP (1 : 1500), SREBF2 (1 : 2000), HMGCR (1 : 1500), LDLR (1 : 1500), PCSK9 (1 : 1500), NR1H3 (1 : 1500), NLRP3 (1 : 2000), ABCG5 (1 : 1500), ABCG8 (1 : 1500), and mouse polyclonal antibodies against *β*-actin (1 : 10000) overnight at 4°C. After washing, the membranes were incubated at room temperature for 45 minutes with appropriate secondary antibodies. Finally, the membranes were treated according to the protocol of the enhanced chemiluminescence detection kit and protein bands were observed by Tanon 4200. The intensities of protein bands were quantified with the Image *J* software and the values normalized to *β*-actin.

### 2.9. Statistical Analysis

All results were presented as mean ± SD. The statistical analysis was performed using SPSS (version 26.0). Differences between the groups were statistically analyzed using one-way analysis of variance (ANOVA). A value of *p* < 0.05 was considered statistically significant. Diagrams are performed by GraphPad Prism version 9.1.

## 3. Results

### 3.1. Changes in Body Weight

**Figure 1:**
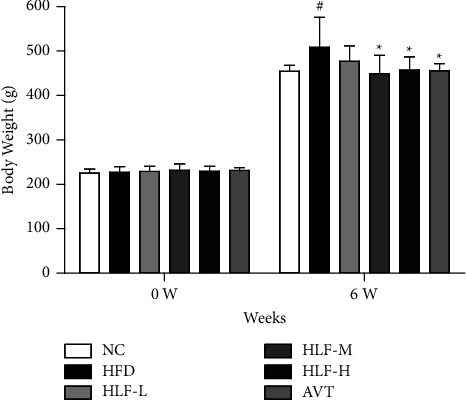
The changes in the body weight of rats before and after HFD feeding (0W: before feeding HFD, 6 W: after 6 weeks of feeding HFD). The data are presented as the mean ± SD (*n* = 8). Note: compared with the HFD group, ^*∗*^*p* < 0.05. Compared with the NC group, ^#^*p* < 0.05.

To evaluate the effect on regulating the levels of blood lipid by HLF, we analyzed the body weight gain of rats for 6 weeks (Figure 1). When compared with the NC group, the body weight of rats in the HFD group had considerably increased by 11.87% after 6 weeks (*p* < 0.05). However, after receiving HLF treatment for four weeks, compared with the HFD group, the body weight of rats in the HLF-M and HLF-H treatment groups was significantly reduced by 11.71% (*p* < 0.05) and 10.04% (*p* < 0.05), respectively.

### 3.2. Serum Lipid Levels in Rats

**Figure 2:**
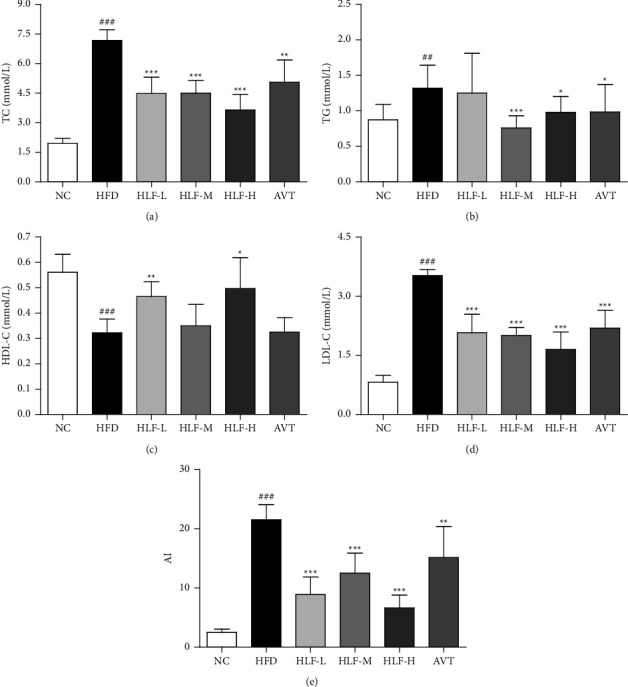
The levels of serum lipids in rats. (a) Serum TC; (b) serum TG; (c) serum HDL-C; (d) serum LDL-C; (e) serum AI. These data are presented as the mean ± SD (*n* = 8). Note: compared with the NC group, ^#^*p* < 0.05; ^##^*p* < 0.01; ^###^*p* < 0.001. Compared with the HFD group, ^*∗*^*p* < 0.05; ^*∗∗*^*p* < 0.01; ^*∗∗∗*^*p* < 0.001.

We used an automatic biochemical analyzer to determine the serum lipid levels in rats. As shown in Figures 2(a)–2(d), the TC, TG, and LDL-C concentrations of the HFD group were significantly increased, and HDL-C levels were significantly decreased compared with the NC group (TC, LDL-C, and HDL-C: *p* < 0.001, TG, *p* < 0.01), indicating the successful establishment of the hyperlipidemia rat model. Compared with the HFD group, the TC and LDL-C levels in the HLF-L, HLF-M, and HLF-H treatment groups were significantly reduced, with TC levels falling by 37.52% (*p* < 0.001), 37.20% (*p* < 0.001), and 49.10% (*p* < 0.001), respectively, LDL-C levels falling by 41.11% (*p* < 0.001), 43.10% (*p* < 0.001), and 53.14% (*p* < 0.001), respectively. Meanwhile, the TG in the HLF-M and HLF-H groups were dramatically decreased by 42.52% (*p* < 0.001) and 25.66% (*p* < 0.05), respectively, and the HDL-C in the HLF-L and HLF-H groups were significantly increased by 44.19% (*p* < 0.01) and 54.26% (*p* < 0.05), respectively, when compared with the HFD group. These findings suggested that HLF can significantly improve the levels of serum lipid in hyperlipidemic rats.

Atherosclerosis is the chronic accumulation of cholesterol-rich plaques within the arteries, which is associated with a range of cardiovascular diseases including peripheral vascular disease, aortic aneurysm, myocardial infarction, and stroke [48]. Atherogenic index (AI, AI = (TC − HDL-C)/HDL-C) is considered as a strong marker to predict the risk of atherosclerosis and coronary heart disease [49]. As shown in Figure 2(e), the AI levels in the HFD group were dramatically raised (*p* < 0.001), compared with the NC group. However, the AI levels in the HLF-L, HLF-M, and HLF-H treatment groups were significantly lowered by 58.68% (*p* < 0.001), 42.00% (*p* < 0.001), and 69.18% (*p* < 0.001), respectively, compared with the HFD group. It is suggested that HLF has potential to inhibit the progression of atherosclerosis in hyperlipidemia rats, which needs further study.

### 3.3. Antioxidant Profiles in Plasma

**Figure 3:**
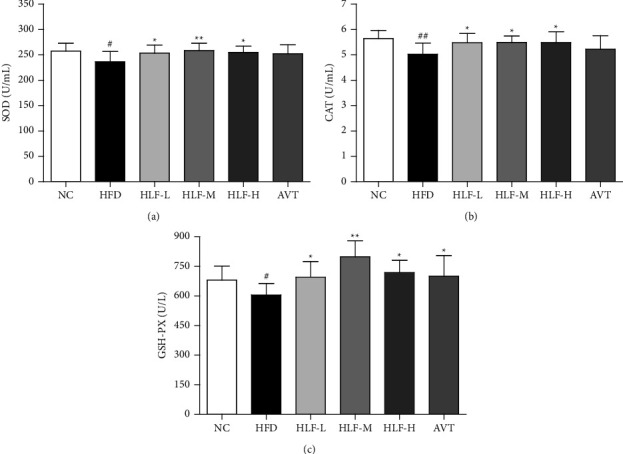
Antioxidant profiles in plasma. (a) Plasma SOD; (b) plasma CAT; (c) plasma GSH-PX. These data are presented as the mean ± SD (*n* = 8). Note: compared with the NC group, ^#^*p* < 0.05; ##*p* < 0.01. Compared with the HFD group, ^*∗*^*p* < 0.05; ^*∗∗*^*p* < 0.01.

There are multiple mechanisms which can be completed through key antioxidants such as SOD, CAT, and GSH-PX in the human body to prevent oxidative stress caused by free radicals [50]. As shown in Figures 3(a)–3(c), compared with the NC group, the activities of plasma SOD, CAT, and GSH-PX in the HFD group were significantly decreased (*p* < 0.05 or *p* < 0.01), illustrating that the oxidative stress response of rats fed the high-fat diet were aggravated. While the activities of SOD, CAT, and GSH-PX in the HLF-L, HLF-M, and HLF-H treatment groups were significantly enhanced, with SOD being increased by 7.18% (*p* < 0.05), 9.30% (*p* < 0.01), and 7.80% (*p* < 0.05), respectively; CAT being raised by 8.92% (*p* < 0.05), 9.24% (*p* < 0.05), and 9.07% (*p* < 0.05), respectively; and GSH-PX being increased by 14.83% (*p* < 0.05), 31.96% (*p* < 0.01), and 18.75% (*p* < 0.05), respectively, compared with the HFD group, which manifested that HLF could significantly inhibit the oxidative stress response in hyperlipidemic rats.

### 3.4. Anti-Inflammatory in Plasma and Intestinal Tissue

**Figure 4:**
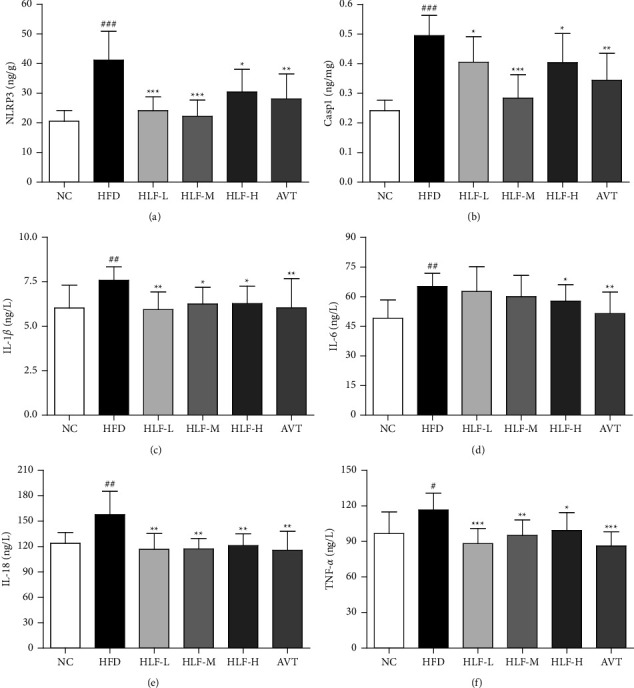
Anti-inflammatory in plasma and intestine tissue. (a) Intestine NLRP3; (b) intestine casp1; (c) plasma IL-1*β*; (d) plasma IL-6; (e) plasma IL-18; (f) plasma TNF-*α*. These data are presented as the mean ± SD (*n* = 8). Note: compared with the NC group, ^#^*p* < 0.05; ^##^*p* < 0.01; ###*p* < 0.001. Compared with the HFD group, ^*∗*^*p* < 0.05; ^*∗∗*^*p* < 0.01; ^*∗∗∗*^*p* < 0.001.

We measured the levels of proinflammatory cytokines, such as plasma IL-1*β*, IL-6, IL-18, TNF-*α*, Casp1, and NLRP3 in the intestinal tissue of rats to better understand the anti-inflammatory effects of HLF. As shown in Figures 4(a)–4(f), the levels of NLRP3, Casp1, IL-1*β*, IL-6, IL-18, and TNF-*α* in the HFD group were significantly increased (*p* < 0.05 or *p* < 0.01 or *p* < 0.001), compared with the NC group, which demonstrated that the inflammatory response of rats fed the high-fat diet were exacerbated.

In addition, compared with the HFD group, the NLRP3 in the HLF-L, HLF-M, and HLF-H treatment groups were significantly reduced by 16.97% (*p* < 0.001), 46.02% (*p* < 0.001), and 26.13% (*p* < 0.05), respectively; the Casp1 in the HLF-L, HLF-M, and HLF-H treatment groups were significantly lowered by 5.03% (*p* < 0.05), 42.56% (*p* < 0.001), and 18.52% (*p* < 0.05), respectively; the IL-1*β* in the HLF-L, HLF-M, and HLF-H treatment groups were significantly decreased by 21.72% (*p* < 0.01), 17.63% (*p* < 0.05), and 17.30% (*p* < 0.05), respectively; and the IL-18 in the HLF-L, HLF-M, and HLF-H treatment groups were significantly reduced by 25.98% (*p* < 0.01), 25.75% (*p* < 0.01), and 23.10% (*p* < 0.01), respectively. Meanwhile, the TNF-*α* in the HLF-L, HLF-M, and HLF-H treatment groups were significantly decreased by 24.33% (*p* < 0.001), 18.46% (*p* < 0.01), and 14.79% (*p* < 0.05), respectively, and the IL-6 in the HLF-H treatment group were significantly lowered by 11.38% (*p* < 0.05), when compared with the HFD group. These results suggested that HLF may be able to somewhat inhibit the inflammatory state of hyperlipidemic rats.

### 3.5. Hepatic Morphology (HE Staining)

**Figure 5:**
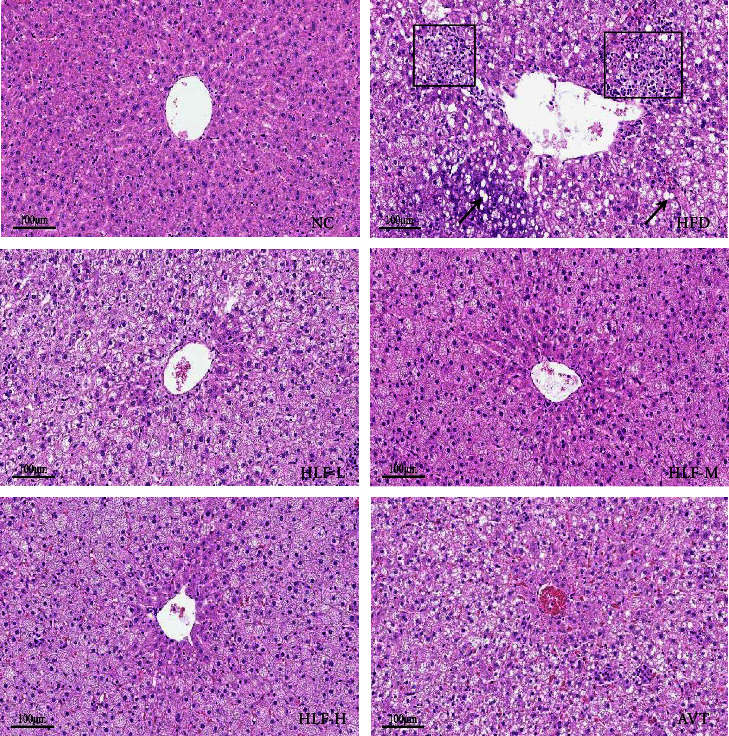
HE staining of the hepatic tissue (200*x*). The black rectangle denotes inflammatory cell infiltration and the black arrow denotes the lipid vacuole.

We used HE staining to analyze the pathological changes of the liver tissue. As shown in Figure 5, the NC group appeared with the normal hepatic lobular structure, normal hepatocytes, no fatty vacuoles in the cytoplasm, and no steatosis or necrosis. While the HFD group showed various degrees of steatosis and a great quantity of lipid vacuoles, the hepatocyte degeneration was mostly round, enlarged in size, and partially was infiltrated by inflammatory cells, which indicated that the high-fat diet induced hepatic steatosis in rats. The three dosages of HLF significantly decreased lipid droplets and lessened the infiltration of inflammatory cells in the liver to different degrees, especially in the HLF-M group, which manifested that HLF could ameliorate the accumulation of lipid droplets and inhibit inflammation in hepatic of rats fed high-fat diet.

### 3.6. Intestinal sIgA

**Figure 6:**
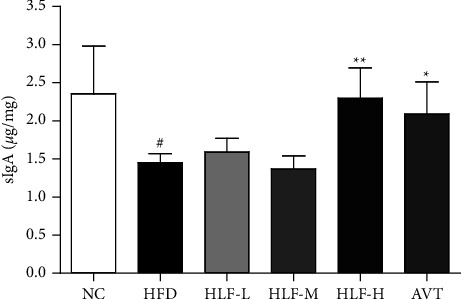
Effects of HLF on the levels of intestinal sIgA in rats with hyperlipidemia. These data are presented as the mean ± SD (*n* = 8). Note: compared with the NC group, ^#^*p* < 0.05; ^##^*p* < 0.01. Compared with the HFD group, ^*∗*^*p* < 0.05; ^*∗∗*^*p* < 0.01.

As shown in Figure 6, the levels of intestinal sIgA in the HFD group were significantly declined (*p* < 0.05), compared with the NC group, suggesting that the intestinal immune functions of rats fed high-fat diet were seriously impaired. Compared with the HFD group, the levels of intestinal sIgA in the HLF-H treatment group were significantly increased by 58.38% (*p* < 0.01), which indicated that HLF could improve the immune function of the gastrointestinal tract by regulating the levels of sIgA.

### 3.7. Plasma TRF and HEPC

**Figure 7:**
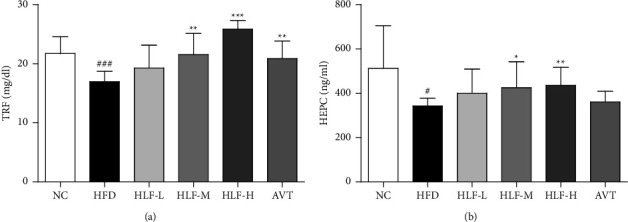
(a) Plasma TRF; (b) plasma HEPC. These data are presented as the mean ± SD (*n* = 8). Note: compared with the NC group, ^#^*p* < 0.05; ^##^*p* < 0.01; ^###^*p* < 0.001. Compared with the HFD group, ^*∗*^*p* < 0.05; ^*∗∗*^*p* < 0.01; ^*∗∗∗*^*p* < 0.001.

Our study showed that the levels of plasma TRF and HEPC in the HFD group of rats were significantly reduced (*p* < 0.05 or *p* < 0.001) compared with the NC group (Figure 7), suggesting that the high-fat diet could lower the levels of plasma iron and increase iron accumulation and deposition in the liver, leading to dysregulation of iron metabolism. Notably, the levels of plasma TRF and HEPC in the HLF-M and HLF-H treatment group were significantly raised, with TRF being increased by 27.17% (*p* < 0.01) and 52.44% (*p* < 0.001), respectively, and HEPC being grown by 23.99% (*p* < 0.05) and 26.95% (*p* < 0.01), respectively, which demonstrated that HLF could modulate the disorder of body iron metabolism.

### 3.8. Species Annotation and Assessment

**Figure 8:**
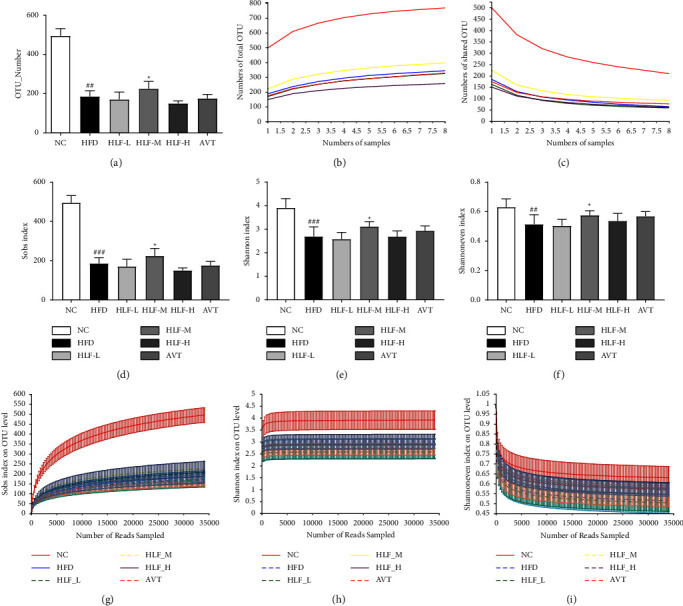
(a) The number of OTUs in each group. (b) Pan species analysis: observations increase in the number of total species with increasing number of samples. (c) Core species analysis: for observing a decrease in the number of shared OTUs as the number of samples increases. (d)–(f) The Sobs, Shannon, and Shannoneven indices of gut microbes in each group. (g)–(i) The rarefaction curve of each dimension. The value is presented as an average ± S.E. (*n* = 8). Differences were assessed by Student's test and denoted as follows: ^#^*p* < 0.05, ^##^*p* < 0.01 (vs. NC); ^*∗*^*p* < 0.05; ^*∗∗*^*p* < 0.01 (vs. HFD).

Annotations and species assessments include primarily OTU (operational classification unit) analysis, alpha diversity analysis, and rarefaction curve analysis. In this study, we conducted 16S rRNA sequences of 48 fecal microbiota samples for 2,425,668 high-quality sequences following quality control. The mean sequence length was 410. The sequence length was mainly distributed in 420∼440 bp, followed by 400∼420 bp. The NC group showed significant differences (*p* < 0.05) in the number of OTUs compared to the rest of HFD. While compared with the number of OTUs in the HFD, only the HLF-M showed a significant difference (*p* < 0.05) (Figure 8(a)). In addition, pan/core analysis showed the total number of species in each increased gradually with increasing sample size, and the number of core species in each tended to remain stable with increasing sample size, indicating an adequate sample size for this experiment (Figures 8(b) and 8(c)).

Based on the OTU levels, the results of *α*-diversity analysis showed that the indices of Sobs (Figure 8(d)), Shannon (Figure 8(e)), Shannoneven (Figure 8(f)) in the HFD group were significantly lower compared with the NC (*p* < 0.01, or *p* < 0.001). The HLF-M had significantly higher Sobs, Shannon, and Shannoneven indices than the HFD (*p* < 0.05). The rarefaction curve based on OTU levels can be observed, accompanied by a gradual increase in sequence depth, and the curve gradually leveled off, indicating that in terms of community richness (Figure 8(g)), community evenness (Figure 8(h)), community diversity (Figure 8(i)), the sequence depth had substantially covered the bacterial species in the fecal samples and the amount of sequence data was sufficient and stable. This indicated that HFD causes a disturbance in the composition of the gut microbiota and HLF could reverse this situation.

### 3.9. Sample Comparison Analysis

**Figure 9:**
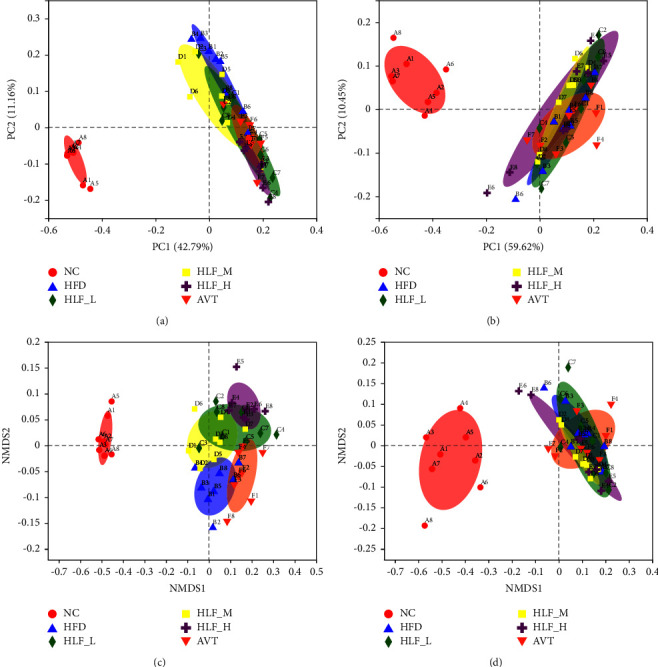
(a) PCoA analysis of unweighted_unifrac (*R* = 0.6844, *p*=0.001000); (b) PCoA analysis of weighted_unifrac (*R* = 0.3512, *p*=0.001000); (c) NMDS analysis of unweighted_unifrac (stress: 0.083, *R* = 0.6844, *p*=0.001000); (d) NMDS analysis of weighted_unifrac (stress: 0.071, *R* = 0.3512, *p*=0.001000). Points of the same color or shape represent samples in different groups. The closer the two sample points are, the more similar the species composition of the two samples is, ANOSIM was used to test the difference between the groups.

To further investigate the similarities or differences between gut microbial compositions, we assessed *β*-diversity at the OTU level using nonmetric multidimensional scaling (NMDS) and principal coordinate analysis (PCoA), with ANOSIM—tests for differences between(Figures 9(a)–9(d), Table 1). The results of PCoA based on unweighted and weighted, as well as NMDS, indicated the microbial composition of NC differed from that of HFD (*p* < 0.01), suggesting the formation of hyperlipidemia altered the composition of the whole gut microbial composition. According to the unweighted analysis, results showed the gut microbial composition of the HLF-H was completely different from that of the HFD (*p* < 0.01), a small portion of the intestinal microbiota of the other treatment groups overlapped with the HFD and only the microbiota of HLF-M showed obvious convergence towards NC. However, the weighted analysis showed that there was increased overlap in the gut microbiota between each treatment and the HFD, and the HLF-H presented a more conspicuous convergence towards NC. Moreover, further analysis using the ANOSIM test revealed that although HLF and AVT showed significant differences in the microbiota compared with HFD (*p* < 0.05), HLF-H had the strongest explanation for the difference from the HFD (*R* = 0.8114).

### 3.10. Species Difference Analysis

**Figure 10:**
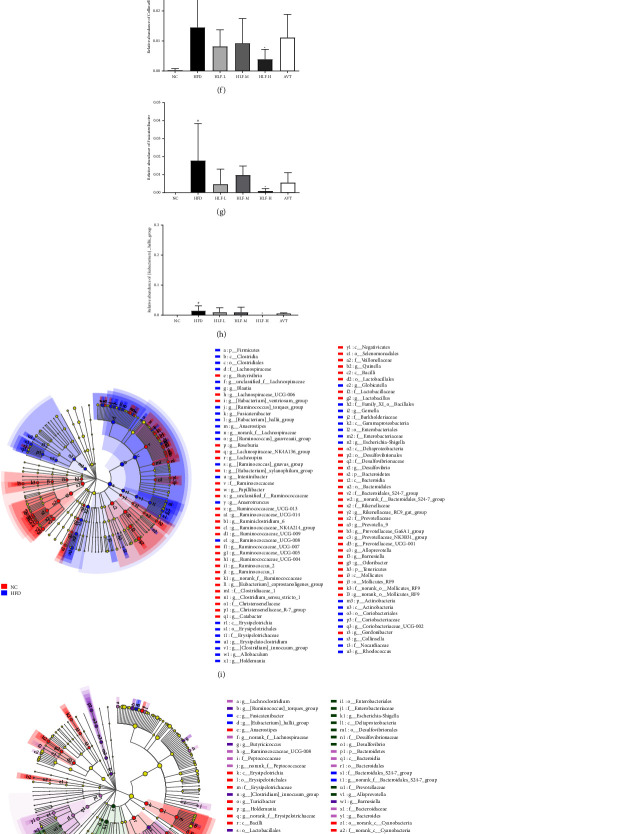
(a) Venn diagram of the OTU number in each group. The number of overlapping parts represents the number of species common to multiple groups, and the number of nonoverlapping parts represents the number of species unique to the corresponding group. (b)–(d) Relative abundance of the gut microbiota at the phylum level and genus level. The ordinate/abscissa is the proportion of species in the sample. The columns of different colors represent different species, and the length of the columns represents the proportion of the species. (e)–(h) Relative abundance of intestinal microbial community members at the genus level in each group. Differences are assessed by the one-way ANOVA test and post hoc using the Tukey–Kramer test which is denoted as follows: ^#^*p* < 0.05, ^##^*p* < 0.01, and ^###^*p* < 0.001 (vs. NC); ^*∗*^*p* < 0.05, ^*∗∗*^*p* < 0.01, and ^*∗∗∗*^*p* < 0.001 (vs. HFD). (i)–(j) Classification branch diagram of LEfSe. Different color nodes represent microbial groups that are significantly enriched in the corresponding groups and have a significant impact on the differences between the groups. The diameter of each circle is directly proportional to the abundance of taxons. (k)–(n) The value is presented as an average ± S.E (*n* = 8).

In this study, we normalized each sample to equal sequencing depth and clustering according to the minimum sample sequence number. Data analysis obtained 906 OTUs with 97% similarity and 149 OTUs in common, detecting 15 phyla, 26 classes, 41 orders, 76 families, 197 genera, and 365 species (Figure 10(a)). Subsequently, we calculated the species richness of each sample at different taxonomic levels, classified the species with an abundance ratio below 0.01 among all samples as others, and averaged the values to calculate within group samples. Five phyla, 15 families, and thirty genera were identified, representing over 0.01% of all samples (Figures 10(b)–10(d)).

By visually displaying the species abundance of each group at different taxonomic levels, we could intuitively show which dominant species each sample contains at the taxonomic level and the relative abundance of the dominant species. At the phylum level, *p_Firmicutes* prevailed in all subjects' gut microbiota, and smaller populations include *p_Bacteroidetes*, *p_Proteobacteria*, *p_Actinobacteria*, and *p_Verrucomicrobia*. Compared with NC, HFD showed a relatively increased abundance of *p_Firmicutes*, *p_Proteobacteria*, and *p_Actinobacteria* and a relatively decreased abundance of *p_Bacteroidetes* and *p_Verrucomicrobia*, indicating the imbalance of gut microbiota dysbiosis in hyperlipidemia. To a certain extent, HLF intervention attenuated the dysbiosis of the gut microbiota. Compared with HFD, the three doses of HLF could increase the relative abundance of *p_Verrucobacteria* and *p_Bacteroidetes*, and reduce the relative abundance of *p_Actinobacteria*. While HLF-L and HLF-H can decrease the relative abundance of *p_Firmicutes*, and HLF-M could decrease the relative abundance of *p_Proteobacteria*. At the family level, *f_Lachnospiraceae* prevailed in all subjects' gut microbiota, and the composition of gut microbiota in each group was partially different. Compared with the NC group, *f_Lachnospiraceae*, *f_Erysipelotrichaceae*, and *f_Enterobacteriaceae* in the HFD group increased significantly and *f_Lactobacillus*, *f_Verrucomicrobiaceae*, and *f_Ruminococcaceae* decreased significantly, while HLF could significantly improve the relative abundance of these species. At the genus level (Figures 10(e)–10(h)), one could clearly see that the structure and relative abundance of the principal microbiota of each group have clearly been altered. We used the significant difference test between the groups to analyze the species with a relative abundance ratio ≥0.01 and evaluate the significance level of the difference in species abundance. Compared with the NC group, the number of *g_Blautia*, *g_Anaerostipes*, and *g_Allobaculum* in the HFD group significantly increased, while the number of *g_Lactobacillus*, *g_Akkermansia*, and *g_Alloprevotella* significantly decreased. It is worth noting that HLF treatment could improve the dysbiosis, among which HLF-H could significantly reduce the relative abundance of *g_Anaerostipes*, *g_Collinsella*, *g_Fusicatenibacter*, and *g_ [Eubacterium]_hallii_group* (*p* < 0.05).

Besides, to further explore differences in the specific gut microbiota among all subjects, we used the linear discriminant analysis effect size (LEfSe) method to recognize the specific altered bacterial phenotypes at each phylogenetic level (from phylum to genus), with linear discriminant analysis (LDA) > 3, ,*p* < 0.05 and multigroup comparison strategy of all-against-all (Figures 10(i)–10(n)). By comparing the significantly different species of NC and HFD, it could be seen that HFD caused a serious imbalance in the composition and relative abundance of intestinal microorganisms. After HLF treatment, the main species of gut microbiota (the proportion of species ≥0.01), such as *p_Verrucomicrobia*, *f_Lactobacillaceae*, *g_Akkermansia*, and *g_Lactobacillus*, changed significantly. Although the results of the LEfSe test were quite different from those of the Tukey–Kramer test, it still showed that HLF could significantly regulate the relative abundance of these species, indicating that these bacteria were associated with the HLF treatment of hyperlipidemia.

### 3.11. Active Ingredients and Targets for HLF

A total of 75 flavonoids compounds in hawthorn leaves were obtained by searching TCMSP, chemistry database, and related literature, as shown in Table 2. By predicting the potential targets of active compounds based on the STITCH and CTD platforms, the 83 targets were finally screened for their possible association with the prevention and treatment of hyperlipidemia in HLF.

### 3.12. Construction and Analysis of PPI Network

**Figure 11:**
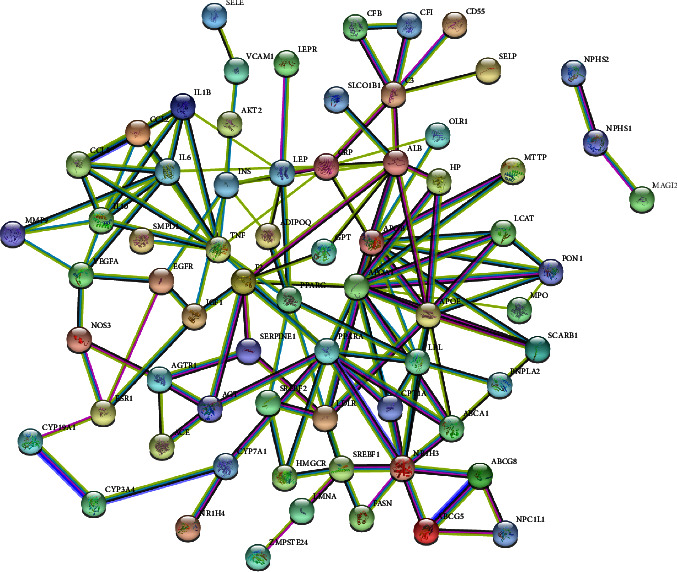
PPI network of HLF. Nodes in the figure represent proteins, and each edge represents a protein-protein interaction relationship, and the more lines represent a greater association. The sky-blue line in the figure represents protein-protein interactions obtained from the created database and the purple line represents experimentally determined protein-protein interactions.

We introduced the abovementioned 83 intersection targets into the string platform, resulting in a column of protein-protein interaction data and a PPI network map. After the minimum interaction threshold was set to “highest confidence” (0.900) and disconnected nodes were hidden, 68 closely linked targets were finally obtained. The PPI network diagram of this study included 68 nodes, 143 edges, and the average node degree was 3.45. The local clustering coefficient was 0.46. The PPI analysis considered that this network to be far more interactive than expected, meaning more protein-protein interactions than would be expected from a set of proteins randomly drawn from the genome with the same size and degree distribution, as shown in Figure 11.

### 3.13. GO Enrichment and KEGG Pathway Analysis

**Figure 12:**
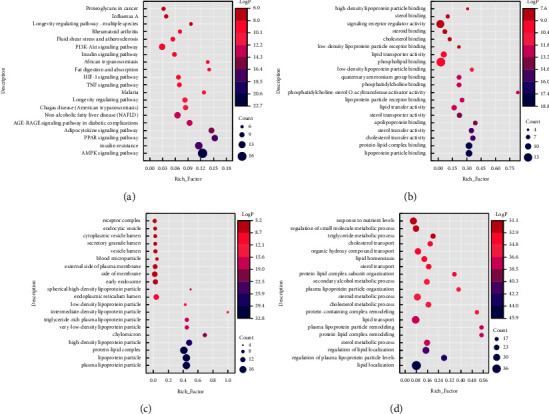
GO enrichment and KEGG pathway analysis. (a) KEGG pathway; (b) molecular function; (c) cellular components; (d) biological processes. The size of the bubbles represents the number of genes in this entry, with cool to warm, representing the −log  *p* value from large to small.

We used the Metascape data platform to perform GO enrichment analysis on HLF antihyperlipidemia-related targets; screened out the top 20 KEGG pathways MF, BP, and CC based on the *p* value; and visualized the results (each bubble chart) using Origin Lab 2021. KEGG pathway analysis revealed 99 pathways related to HSA term, including AGE-RAGE signaling pathway in diabetic complications, insulin resistance, and AMPK signaling pathway. MF analysis yielded 92 results, mainly enriched in cholesterol transfer activity, sterol transfer activity, and lipoprotein particle binding. The result of CC enrichment is 55, mainly including vesicle lumen, plasma lipoprotein particle, lipoprotein particle, and so on; BP enrichment results obtained 579, mainly related to lipid localization, regulation of lipid localization, lipid storage, and other biological processes, as shown in Figures 12(a)–12(d).

### 3.14. Network of HLF Active Ingredients—Antihyperlipidemic Targets-Biological Processes

**Figure 13:**
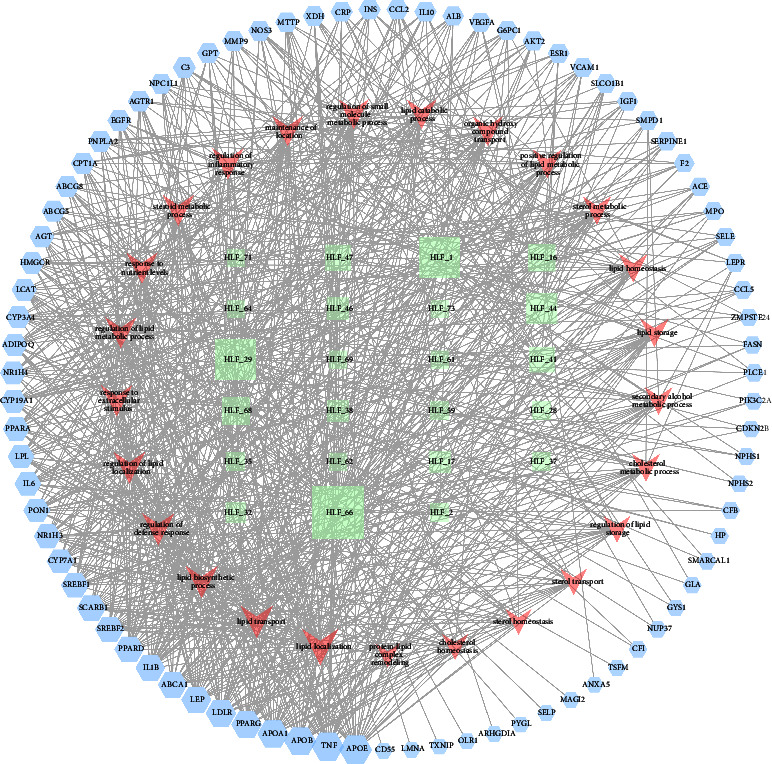
Network of HLF active ingredients—antihyperlipidemic targets-biological processes. In the output network, nodes with different colors represent compounds, targets, and action pathways. Circular nodes indicate the active ingredients associated with HLF, ortho hexagonal nodes symbolize targets of action, and inverted triangles represent the top 25 biological processes.

In this study, the compound, target, and biological processes' information obtained above were imported to Cytoscape 3.8.2 software to construct the “HLF active ingredient antihyperlipidemia target pathway network” (Figure 13). The network contained 131 nodes, 843 edges, and the network concentration was 0.396. It was predicted that quercetin was the main component of antihyperlipidemia in HLF, followed by (+)-catechin, epicatechin, and so on. Taken together the KEGG analysis and the degree values, we supposed that the targets of HLF against hyperlipidemia might be related to the biosynthesis, transport, and homeostasis regulation system of several lipids, including cholesterol, steroids, and fatty acids, and the targets were mainly enriched in APOE, LDLR, PPARG, and so on.

### 3.15. The Potential Drug Targets for Antihyperlipidemia by HLF

**Figure 14:**
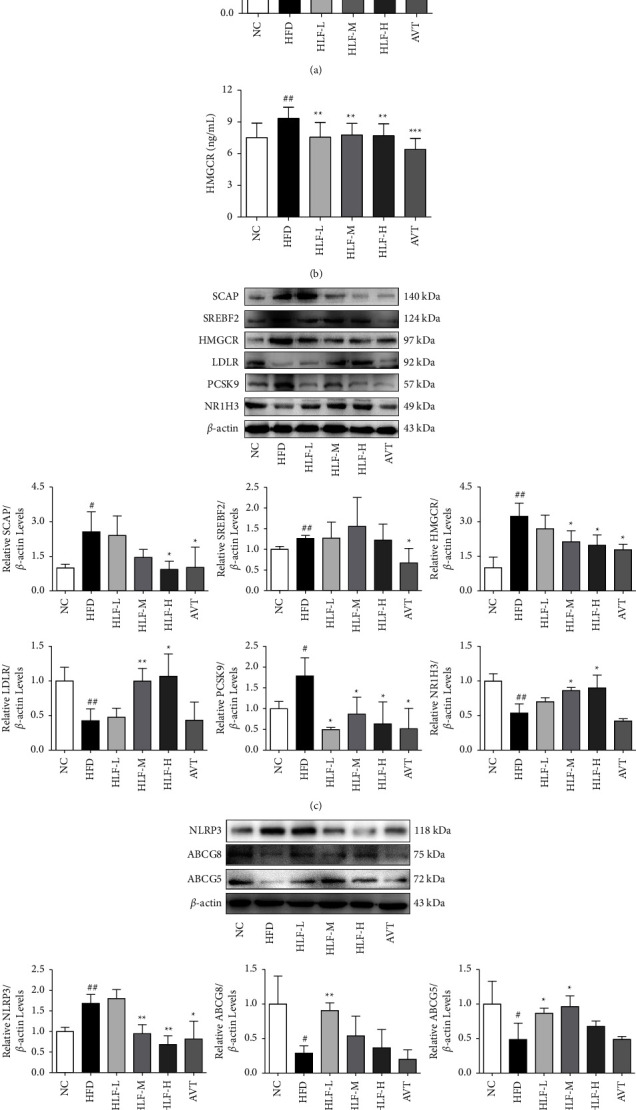
The potential drug targets for antihyperlipidemia by HLF. (a) The level of CYP7A1 in the intestinal tissue; (b) the level of HMGCR in the plasma level; (c) the expression of related targets in the liver; (d) the expression of related targets in the intestine. These data are presented as the mean ± SD (*n* = 3). Note: compared with the NC group, ^#^*p* < 0.05; ^##^*p* < 0.01. Compared with the HFD group, ^*∗*^*p* < 0.05; ^*∗∗*^*p* < 0.01.

Based on the network pharmacology, literature query, and results of our previous study, we validated the expression of CYP7A1, HMGCR, SCAP, and other targets in HFD rats and the effect of HLF on them. The ELISA test results showed that compared with the NC group (Figures 14(a) and 14(b)), the level of CYP7A1 in HFD rats was decreased significantly (*p* < 0.01), and the HMGCR level was increased significantly (*p* < 0.01). Compared with the HFD group, the levels of CYP7A1 in the HLF-L, HLF-M, and HLF-H treatment group were significantly increased (*p* < 0.05), while the HMGCR levels in the three dosages of HLF were significantly reduced (*p* < 0.01).

As shown in Figures 14(c) and 14(d), the protein expression profiles of SCAP, PCSK9, HMGCR, SREBF2, and NLRP3 (*p* < 0.05 or *p* < 0.01) in the liver or intestine of HFD rats were significantly upregulated, while the expressions of LDLR, NR1H3, ABCG5, and ABCG8 were significantly downregulated (*p* < 0.05 or *p* < 0.01) compared with the NC group. Compared with the HFD group, the protein expression profiles of SCAP, HMGCR, and NLRP3 were downregulated by HLF (*p* < 0.05 or *p* < 0.01). Meanwhile, the expression profiles of PCSK9 in the three dosages of the HLF group were significantly decreased (*p* < 0.05). However, the protein expression profiles of ABCG5, ABCG8, LDLR, and NR1H3 were upregulated by HLF (*p* < 0.05 or *p* < 0.01). Although HLF did not significantly inhibit the expression of SREBF2 compared with the HFD group, a very clear decreasing trend was still seen. These results indicated that the mechanism of HLF treatment for hyperlipidemia may be related to the regulation of cholesterol biosynthesis, metabolism, and transport.

### 3.16. Correlation Analysis of Intestinal Microbes

**Figure 15:**
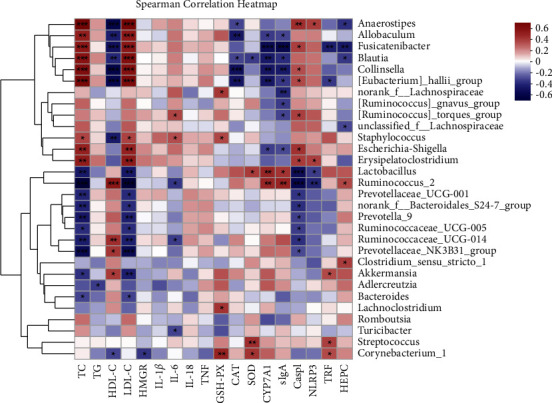
Correlation heatmap graph. The *X*- and *Y*-axes are clinical factors and species, respectively, for which the correlation *R*-values and *p* values are obtained by calculation. The *R*-values are shown in different colors in the figures. If the *p* value is less than 0.05, it is marked with ^*∗*^. The legend on the right is the color interval of different *R*-values. The value is presented as an average ± S.E. (*n* = 8). Differences were assessed by Spearman and denoted as follows: ^*∗*^*p* < 0.05; ^*∗∗*^*p* < 0.01; ^*∗∗∗*^*p* < 0.001.

Based on the bacteria at the genus level, we used Spearman's test to analyze the relationship between bacteria and each indicator in plasma and serum (Figure 15). In blood lipid levels, the abundance of *g_Lactobacillus* and *g_Akkermansia* were significantly negatively correlated with the levels of LDL-C and TC, but significantly positively correlated with the levels of HDL-C. The abundances of *g_Anaerostipes*, *g_[Eubacterium]_hallii_group*, *g_Collinsella*, and *g_Fusicatenibacter* were significantly positively correlated with the levels of LDL-C and TC, and negatively correlated with the levels of HDL-C. In terms of cholesterol metabolism, CYP7A1 was a significant target with a significant correlation to bacterial production and a significant positive correlation to the abundance of *g_Lactobacillus* and a significant negative correlation to the abundance of *g_[Eubacterium]_hallii_group*, *g_Collinsella*, and *g_Fusicatenibacter*. In terms of antiinflammation, the abundance of *g_Anaerostipes* was significantly positively correlated with the level of NLRP3, but the abundance of *g_Lactobacillus* was significantly negatively correlated with the level of NLRP3. In terms of immunity, Casp1 and sIgA were important indicators that are significantly related to bacteria. The abundances of *g_[Eubacterium]_hallii_group*, *g_Anaerostipes*, *g_Collinsella*, and *g_Fusicatenibacter* were significantly positively correlated with the level of Casp1 and negatively correlated with the level of sIgA. However, the correlation of *g_Lactobacillus* was just the opposite of these bacteria. In terms of antioxidants, the abundance of *g_Collinsella*, *g_[Eubacterium]_hallii_group*, and *g_Anaerostipes* was significantly negatively correlated with the level of CAT, while the abundance of *g_Lactobacillus* was significantly positively correlated with the level of SOD. In terms of iron metabolism, *g_Anaerostipes* and *g_Fusicatenibacter* were negatively correlated with the level of HEPC, while *g_Fusicatenibacter* and *g_[Eubacterium]_hallii_group* were negatively correlated with the level of TFR and *g_Akkermansia* was positively correlated with the level of TFR.

## 4. Discussion

Hyperlipidemia, whose pathogenesis is very complex, is accompanied by the ascent of serum TC, TG, and LDL-C and the decrease of HDL-C levels, which is inseparable from the physiopathological processes, and cholesterol accumulation caused by any factor can lead to hyperlipidemia and aggravate the occurrence as well as development of cardiovascular disease [51, 52]. Increased intestinal cholesterol absorption or increased liver cholesterol biosynthesis can easily cause the accumulation of cholesterol in the body, promoting the activation of NLRP3 inflammasome and triggering the expression of inflammatory factors such as interleukin-1 beta (IL-1*β*), interleukin-18 (IL-18), and tumor necrosis factor-*α* (TNF-*α*) [53, 54]. The high concentrations of cholesterol or cholesterol crystals can promote the activation of NLRP3 inflammasome to start inflammation associated with hyperlipidemia or atherosclerosis [55, 56]. In this study, HLF reduced the serum TC, TG, and LDL-C levels and increased HDL-C levels in hyperlipidemia model rats, improving lipid profiles. It is also shown that HLF could attenuate the liver tissue swelling and improve inflammatory cell infiltration or fatty lesions. Moreover, HLF could significantly reduce the levels of NLRP3, caspase-1, IL-1*β*, IL-6, IL-18, and TNF-*α* and increase the activities of plasma SOD, CAT, and GSH-PX, which suggested that HLF could effectively relieve the inflammatory response and oxidative stress induced by hyperlipidemia.

Cholesterol synthesis and metabolism require a large number of enzymes to catalyze, among which 3-hydroxy-3-methylglutaryl-coenzyme A reductase (HMGCR), a key enzyme in cholesterol biosynthesis, is regulated by SCAP/SREBF2 (sterol regulatory element-binding protein cleavage-activating protein/sterol regulatory element-binding protein 2) regulation [57, 58]. When cholesterol is low in the cell, SCAP activates the cleavage of S1P protease and the release of the active fragment, which will activate the expression of the downstream target gene HMGCR, and ultimately contributes to increase cellular cholesterol uptake instead of endogenous synthesis [59–61]. When cells are high in cholesterol, SCAP inhibits this cleavage reaction, which leads to decrease the expression of the downstream target genes HMGCR [62, 63]. Finally, compared with the endogenous synthesis, it leads to a decrease in cellular cholesterol uptake to maintain cholesterol homeostasis. In this study, HLF could lower the level of HMGCR in plasma and decrease the protein expression profiles of HMGCR and SCAP in HFD rats, which demonstrated that HLF could inhibit cholesterol biosynthesis and improve the lipid-lowering activity.

Low-density lipoprotein receptor (LDLR) is the liver surface receptor of LDL, responsible for removing LDL-C from human blood. After binding to LDL particles, LDLR is internalized into clathrin-coated pits and then transports LDL from the cytoplasm to the lysosome for degradation [64]. Proprotein convertase subtilisin/kexin type 9 (PCSK9) is a soluble protein and a ligand for LDLR. Extracellular PCSK9 binds to LDLR through protein-protein interactions and directly enters the lysosome as a PCSK9-LDLR complex for destruction, inhibiting LDLR recirculation and lowering plasma LDL-C levels [65, 66]. The conversion of cholesterol into bile acid (BA) in the liver is its main metabolic pathway. Cholesterol 7*α*-hydroxylase (CYP7A1) is the rate limiting enzyme in the conversion of cholesterol to Bas [67]. The expression of CYP7A1 is regulated by the liver *X* receptor alpha (NR1H3), and its activity determines the rate of BA synthesis [68]. In addition, ATP-binding cassette subfamily *G* member 5 and 8 (ABCG5/G8) are a heterodimeric complex, mainly on the tubule membrane of hepatocytes and the apical membrane of intestinal cells, which can regulate cholesterol metabolism by promoting the excretion of liver cholesterol into bile and reducing the absorption of cholesterol by the intestine [69, 70]. The expression of ABCG5/G8 can prevent liver fat accumulation by reducing cholesterol concentration and fatty acid intake [71]. In the liver, the main regulator of ABCG5 and ABCG8 mRNA expression is NR1H3, and NR1H3 promotes cholesterol excretion by regulating ABCG5/G8 transporters [72, 73]. In this study, HLF could significantly downregulate the expression of PCSK9; increase the level of CYP7A1 in the intestinal tissue; and upregulate the expression of LDLR, NR1H3, ABCG5, and ABCG8 in HFD rats to decrease the intestinal absorption of cholesterol, promoting cholesterol excretion.

HEPC is the most master regulator of the body iron metabolism, and increased uptake of iron by the liver leads to increased production and secretion of hepcidin, which regulates iron metabolism by inhibiting ferroportin located in intestinal enterocytes and macrophages. Contrary to the expected results, plasma HEPC and TRF levels were both decreased in the studies in which we used high-fat fed animals, a situation consistent with the experimental results of Ye [74]. This may be caused by dysregulation of iron metabolism due to hepatic impairment, as a manifestation of decreased hepcidin is also seen in patients with chronic liver disease [75]. In addition, in previous studies, patients with dyslipidemia and atherosclerosis also presented significant reductions in TRF levels [76, 77]. In this study, HLF was able to significantly increase plasma TRF and HEPC levels, which suggested that HLF could regulate the disorder of the body iron metabolism.

Structural variations in the gut microbiome are associated with the health of the host, and studies on the composition of the gut microbes are helpful for the diagnosis and treatment of hyperlipidemia [78, 79]. According to the results of the difference and LEfSe test for each group, the species in the HLF group with significant differences were mostly concentrated in *p_Firmicute*, 、*p_Actinobacteria*, and *p_Verrucomicrobia*. The *p_Firmicutes* were mainly enriched in *f_Lachnospiraceae*. *F_Lachnospiraceae* produce high amounts of short-chain fatty acids, which is the largest butyrate producing group of Firmicutes [80, 81]. It has turned out to be important to maintain the metabolic health of the gut microbiota and the stability of the internal environment [82, 83]. This microbial imbalance may be related to the changes in fatty acid levels in the gut [84, 85]. Its abundance is closely related to glycolipid metabolism [86, 87], host immune activation [88], and inflammatory response [89], which in turn affects the level of bile acid [90]. Specifically, HLF had significant effects on *g_Lactobacillus*, *g_Anaerostipes*, *g_[Eubacterium]_hallii_group*, and *g_Fusicatenibacter* belonging to *f_Lachnospiraceae* in the gut microbes of hyperlipidemia rats, indicating that HLF may affect the levels of short-chain fatty acids to realize the treatment of hyperlipidemia by regulating the relative abundance of these gut microbe.

*g_Akkermansia* is an important part of *p_Verrucomicrobia*, which can use mucin as the sole carbon and nitrogen source and release free forms of sulfate from mucin fermentation, resulting in improved host metabolism [91, 92]. In the metabolic syndrome, obesity and hypertriglyceridemia were most strongly associated with *g_Akkermansia*; followed by reduced HDL cholesterol, hypertension, and hyperglycemia; and increasing *g_Akkermansia* abundance can reverse the effects of a high-fat, high-cholesterol diet [93, 94]. Studies have shown that the mechanism of adjusting the relative abundance of *g_Akkermansia* in the treatment of metabolic diseases may be related to its ability to stimulate GLP-1 secretion [95, 96], promote 5-HT biosynthesis, and intestinal stem cell-mediated epithelial development [97, 98]. *g_Collinsella* belongs to *f_Coriobacteriaceae* and *p_Actinobacteria*, which produces lactate, formate, and butyrate [99] can modify host bile acids and influence metabolism by altering intestinal cholesterol absorption, reducing hepatic glycogen production and increasing triglyceride synthesis [100–102]. In terms of inflammation, *g_Collinsella* also increases intestinal permeability, decreases the expression of tight junction protein in epithelial cells, and induces the expression of IL-17 [103–105]. At present, the molecular mechanism by which *g_Collinsella* affects host metabolism is not yet clear [106]. It is certain that *g_Collinsella* is involved in the progression of ulcerative colitis [107], hyperlipidemia [108], and diabetes [109]. A recent study found that a lower dietary fiber intake may lead to an increased abundance of *g_Collinsella*. A structured weight loss program could significantly reduce the abundance of *g_Collinsella* in patients [110].

Anaerostipes is closely related to eating habits and inflammation in obese people, but the precise mechanism is unclear [103, 111]. It is reported that by promoting propionate formation via inositol or phytate, anaerostipes may lower the risk of metabolic disorders [112]. These species, which can use a variety of substrates as well as lactate and acetate to create butyrate, are among the most effective lactate consumers in the human colon [113, 114]. This is essential for maintaining healthy intestinal barrier function. This is essential for maintaining healthy intestinal barrier function [115].

Spearman's correlation analysis further proved that these genera were closely connected with the regulatory effects of HLF on lipid metabolism, cholesterol transport, metabolism, immunity, inflammation, and oxidative stress. The present study suggested that the mechanism of HLF in treating hyperlipidemia may be related to regulate significantly the relative abundance of some bacteria, such as *g_Lactobacillus*, *g_Anaerostipes*, *g_[Eubacterium]_hallii_group*, *g_Fusicatenibacter*, *g_Akkermansia*, *g_Collinsella*, and other bacteria. These dominant bacterial genera altered by HLF showed strong correlations with the hyperlipidemia-related metabolic parameters in HFD-fed rats.

## 5. Conclusion

**Figure 16:**
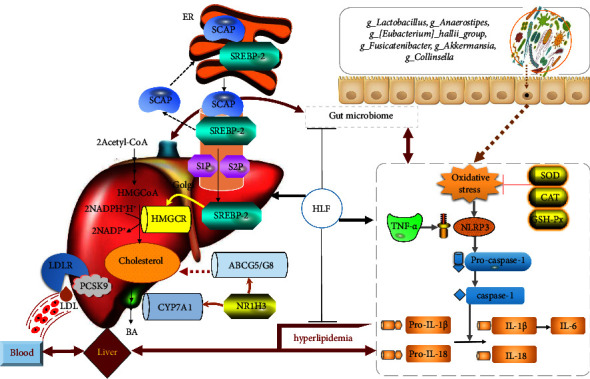
The effects of hawthorn leaf flavonoids on the physiopathological processes of hyperlipidemia through modulating lipid metabolism and gut microbiota.

In conclusion, HLF could play a role in maintaining the normal level of blood lipids in a great deal of ways (Figure 16). We have shown that HLF could improve disorders of lipid metabolism by inhibiting the absorption of intestinal cholesterol and promoting cholesterol excretion. Meanwhile, HLF played an important role in controlling the levels of cholesterol synthesis. In addition, HLF could effectively alleviate oxidative stress and inflammatory response induced by hyperlipidemia. Furthermore, HLF could also regulate the relative abundance of gut microbiota, such as *g_Lactobacillus*, *g_Anaerostipes*, *g_[Eubacterium]_hallii_group*, *g_Fusicatenibacter*, *g_Akkermansia*, *g_Collinsella*, and other bacteria, which may be an effective way to modulate lipid metabolism.

## Figures and Tables

**Table 1:** Result of the analysis of similarities (ANOSIM, *n* = 8).

Groups	Unweighted_unifra		Weighted_unifra	
	*R* statistic	*p* value	*R* statistic	*p* value
NC vs. HFD	1	0.001	0.9911	0.001
HFD vs. HLF-L	0.3242	0.012	-0.0413	0.608
HFD vs. HLF-M	0.2444	0.021	0.0603	0.194
HFD vs. HLF-H	0.8114	0.001	0.0497	0.195
HFD vs. AVT	0.4715	0.003	0.1099	0.097

**Table 2:** HLF for compound information.

Molecule name	Rename	Formula	MW (g/mol)
(+)-Catechin	HLF_1	C15H14O6	290.27
(+)-Taxifolin	HLF_2	C15H12O7	304.25
(+)-Taxifolin 3-O-xylopyranoside	HLF_3	C20H20O11	436.37
(+)-Taxifolin3-O-arabinopyranoside 3-O-arabinopyranoside	HLF_4	C20H20O11	436.37
2″-O-acetylvitexin	HLF_5	C23H22O11	474.41
3‴, 4‴-di-O-acetyl-2″-O-*α*-rhamnosylvitexin	HLF_6	C31H38O16	666.62
3″-O-acetylvitexin	HLF_7	C23H22O11	474.41
3-O-*β*-D-6″-acetylglucopyranoside quercetin	HLF_8	C23H22O13	506.41
4‴-O rhamnosylrutin	HLF_9	C33H40O20	756.66
5-hydroxyauranetin	HLF_10	C20H20O8	388.37
6-C-glucoside-8-C-xylsoyl apigenin	HLF_11	C26H28O14	564.49
7-O-rhamnogalactoside quercetin	HLF_12	C27H30O16	610.52
8-C-*β*-D-(2″-O-acetyl)-glucofuranosyl apigenin	HLF_13	C23H22O11	474.41
8-Methoxykaempferol3-neohesperidoside	HLF_14	C28H32O16	624.54
Acetylvitexin 2″-O-rhamnoside	HLF_15	C29H32O15	620.56
Apigenin	HLF_16	C15H10O5	270.24
Bioquercetin	HLF_17	C27H30O16	610.52
Catiguanin B	HLF_18	C25H22O10	482.44
Cinchonain ib	HLF_19	C24H20O9	452.41
Cinchonain IIb	HLF_20	C39H32O15	740.66
Crataegunin A	HLF_21	C25H22O9	466.44
Crataegunin B	HLF_22	C24H20O9	452.41
Crataegunin C	HLF_23	C25H24O10	484.45
Crataegunin D	HLF_24	C24H20O9	452.41
Crataequinone B	HLF_25	C12H6O6	246.17
Cratenacin	HLF_26	C29H32O15	620.56
Crateside	HLF_27	C20H20O11	436.37
Ent-epicatechin	HLF_28	C15H14O6	290.27
Epicatechin	HLF_29	C15H14O6	290.27
Epicatechin-(4*β*⟶6)-epicatechin-(4*β*⟶8)-epicatechin	HLF_30	C45H38O18	866.77
Epicatechin-(4*β*⟶8)-epicatechin-(4*β*⟶6)-epicatechin	HLF_31	C45H38O18	866.77
Eriodectyol	HLF_32	C15H12O6	288.25
Eriodictyol-5,3′-di-glucoside	HLF_33	C27H32O16	612.53
Herbacetin	HLF_34	C15H10O7	302.24
Hyperin	HLF_35	C21H20O12	464.38
Isoorientin	HLF_36	C21H20O11	448.38
Isoquercitrin	HLF_37	C21H20O12	464.38
Isorhamnetin	HLF_38	C16H12O7	316.26
Isoschaftoside	HLF_39	C26H28O14	564.49
Apigenin-C-hexoside	HLF_40	C21H20O10	432.38
Kaempferol	HLF_41	C15H10O6	286.24
Kaempferol 3-neohesperidoside	HLF_42	C27H30O15	594.52
Leucodelphinidin	HLF_43	C15H14O8	322.27
Luteolin	HLF_44	C15H10O6	286.24
Methoxykaempferol-O-glucoside	HLF_45	C21H20O11	448.38
Myricetin	HLF_46	C15H10O8	318.24
Naringenin	HLF_47	C15H12O5	272.25
Naringenin-5,7-di-glucoside	HLF_48	C27H32O15	596.53
Neoisoschaftoside	HLF_49	C26H28O14	564.49
Neoschaftoside	HLF_50	C26H28O14	564.49
Orientin	HLF_51	C21H20O11	448.38
Pinnatifinoside *A*	HLF_53	C23H20O10	456.4
Pinnatifinoside *B*	HLF_54	C23H20O10	456.4
Pinnatifida *C*	HLF_55	C21H18O9	414.36
Pinnatifida *D*	HLF_52	C23H20O10	456.4
Pinnatifinoside *I*	HLF_56	C23H20O10	456.4
Proanthocyanidin *A*2	HLF_57	C30H24O12	576.5
Procyanidin *B*1	HLF_58	C30H26O12	578.52
Procyanidin dimer *B*2	HLF_59	C30H26O12	578.52
Procyanidin *B*4	HLF_60	C30H26O12	578.52
Procyanidin *B*5	HLF_61	C30H26O12	578.52
Procyanidin *C*1	HLF_62	C45H38O18	866.77
Procyanidin *E*1	HLF_63	C75H62O30	1443.3
Procyanidin tetramer	HLF_64	C60H50O24	1155.02
Propelargonidin dimer	HLF_65	C30H26O11	562.52
Quercetin	HLF_66	C15H10O7	302.24
Quercetin-3-O-(2,6-di-*α*-l-rhamnopyranosyl)-*β*-d-galactopyranoside	HLF_67	C33H40O20	756.66
Rutin	HLF_68	C27H30O16	610.52
Santin	HLF_69	C18H16O7	344.32
Schaftoside	HLF_70	C26H28O14	564.49
Vitexin	HLF_71	C21H20O10	432.38
Vitexin-2-O-rhamnoside	HLF_72	C27H30O14	578.52
Vitexin-2″-O-glucoside	HLF_73	C27H30O15	594.52
4″-O-glucosylvitexin	HLF_74	C27H30O15	594.52
Vitexin-6″-O-acetyl	HLF_75	C23H22O11	474.41

## Data Availability

The data are available from the corresponding author on reasonable request.

## Abbreviations

ABCG5: ATP-binding cassette subfamily *G* member 5
ABCG8: ATP-binding cassette subfamily *G* member 8
ASCVD: Atherosclerotic and cardiovascular disease
AVT: Atorvastatin
BP: Biological processes
Casp1: Caspase-1
CAT: Catalase
CC: Components
CTD: Comparative toxicogenomics database
CYP7A1: Cholesterol 7*α*-hydroxylase
GSH-Px: Glutathione peroxidase
HDL-C: High-density lipoprotein cholesterol
*H* & *E*: Hematoxylin and eosin
HEPC: Hepcidin
HFD: High-fat diet
HLF: Hawthorn leaf flavonoids
HLF-L: Low-dose group of HLF, 100 mg/kg
HLF-M: Medium-dose group of HLF, 200 mg/kg
HLF-H: High-dose group of HLF, 400 mg/kg
HMGCR: 3-hydroxy-3-methylglutaryl-coenzyme*A* reductase
IL-1*β*: Interleukin-1 beta
IL-6: Interleukin-6
IL-18: Interleukin-18
KW: Kruskal–Wallis
LDA: Linear discriminant analysis
LDL-C: Low-density lipoprotein cholesterol
LDLR: Low-density lipoprotein receptors
LEfSe: Linear discriminant analysis effect size
MF: Molecular functions
NC: Normal control
NLRP3: NOD-like receptor protein 3
NMDS: Nonmetric multidimensional scaling analysis
PCSK9: Proprotein convertase subtilisin/kexin type 9
PCoA: Principal coordinate analysis
PDB: Protein data bank
PPI: Protein-protein interactions
SCAP: Sterol regulatory element-binding protein cleavage-activating protein
SD: Sprague–dawley
SOD: Total superoxide dismutase
TC: Total cholesterol
TCM: Traditional Chinese medicine
TCMSP: Traditional Chinese medicine database and analysis platform
TFR: Transferrin
TG: Triglyceride
TNF-*α*: Tumor necrosis factor-alpha.

## Supplementary Materials

Supplementary Materials Supplementary materials of this article contain the molecular docking diagram of HLF. Autodock tools-1.5.6 software is used to dock the active ingredients and target proteins of HLF, and the conformation with the lowest docking binding energy is selected for docking mode analysis, and PyMOL software is used for molecular docking mapping and display. Pharmacophore analysis was performed using the Ligplot + program to evaluate hydrogen bonds and van der Waals interaction residues.
