# Supplementary material for: The Hypolipidemic Effect of Hawthorn Leaf Flavonoids through Modulating Lipid Metabolism and Gut Microbiota in Hyperlipidemic Rats
Source: Evid Based Complement Alternat Med. 2022 Nov 15;2022:3033311. doi: 10.1155/2022/3033311 (PMC9681556; doi:10.1155/2022/3033311)
Supplement: Supplementary Materials — of this article contain the molecular docking diagram of HLF. Autodock tools-1.5.6 software is used to dock the active ingredients and target proteins of HLF, and the conformation with the lowest docking binding energy is selected for docking mode analysis, and PyMOL software is used for molecular docking mapping and display. Pharmacophore analysis was performed using the Ligplot + program to evaluate hydrogen bonds and van der Waals interaction residues. [file 3033311.f1.zip › phylum level.pdf]

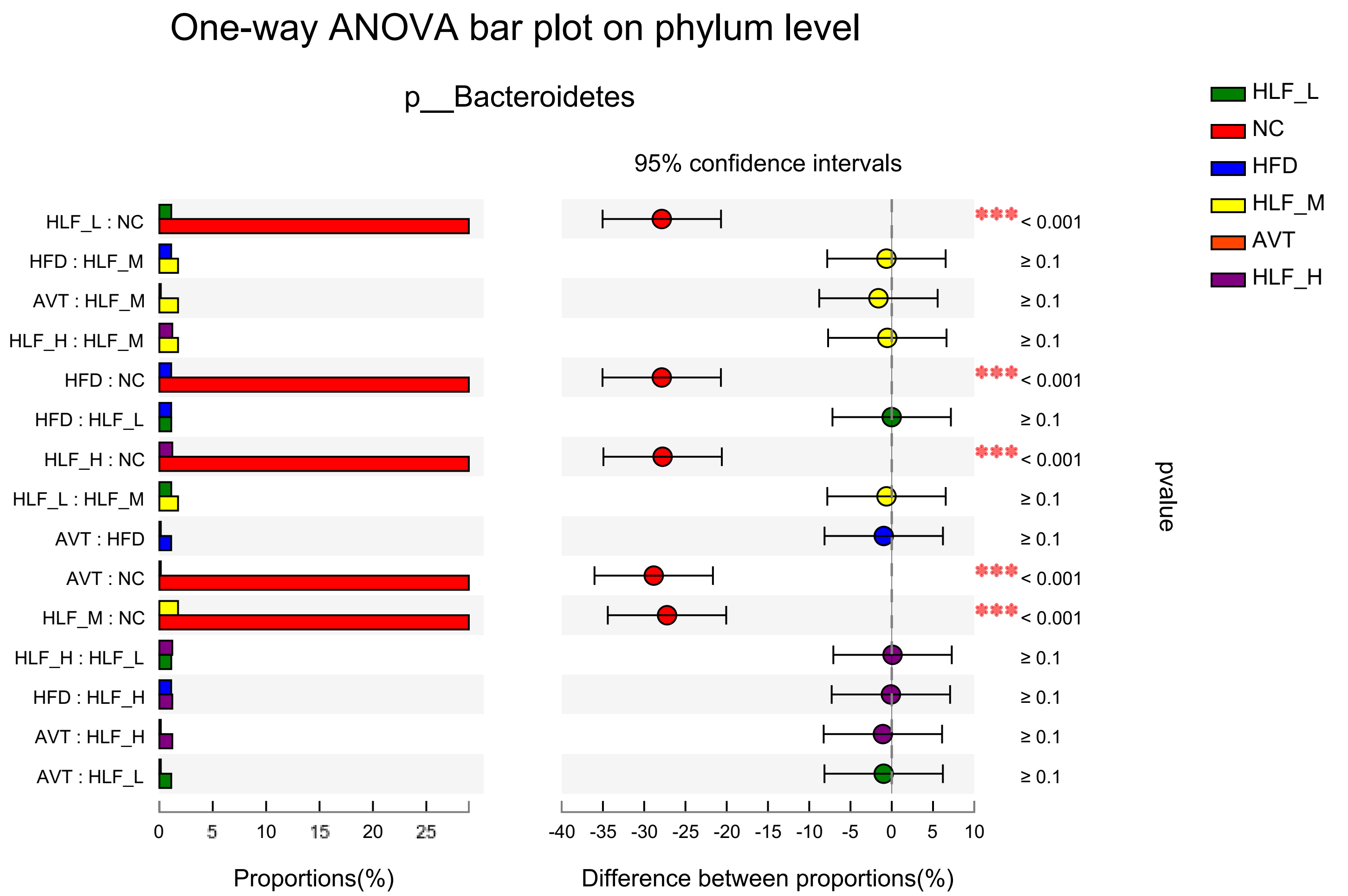
One-way ANOVA bar plot on phylum level
p__Bacteroidetes
95% confidence intervals
HLF_L
NC
HFD
HLF_M
AVT
HLF_H
HLF_L : NC
HFD : HLF_M
AVT : HLF_M
HLF_H : HLF_M
HFD : NC
HFD : HLF_L
HLF_H : NC
HLF_L : HLF_M
AVT : HFD
AVT : NC
HLF_M : NC
HLF_H : HLF_L
HFD : HLF_H
AVT : HLF_H
AVT : HLF_L
*** < 0.001
≥ 0.1
≥ 0.1
≥ 0.1
*** < 0.001
≥ 0.1
*** < 0.001
≥ 0.1
≥ 0.1
*** < 0.001
*** < 0.001
≥ 0.1
≥ 0.1
≥ 0.1
≥ 0.1
pvalue
0 5 10 15 20 25
Proportions(%)
-40 -35 -30 -25 -20 -15 -10 -5 0 5 10
Difference between proportions(%)

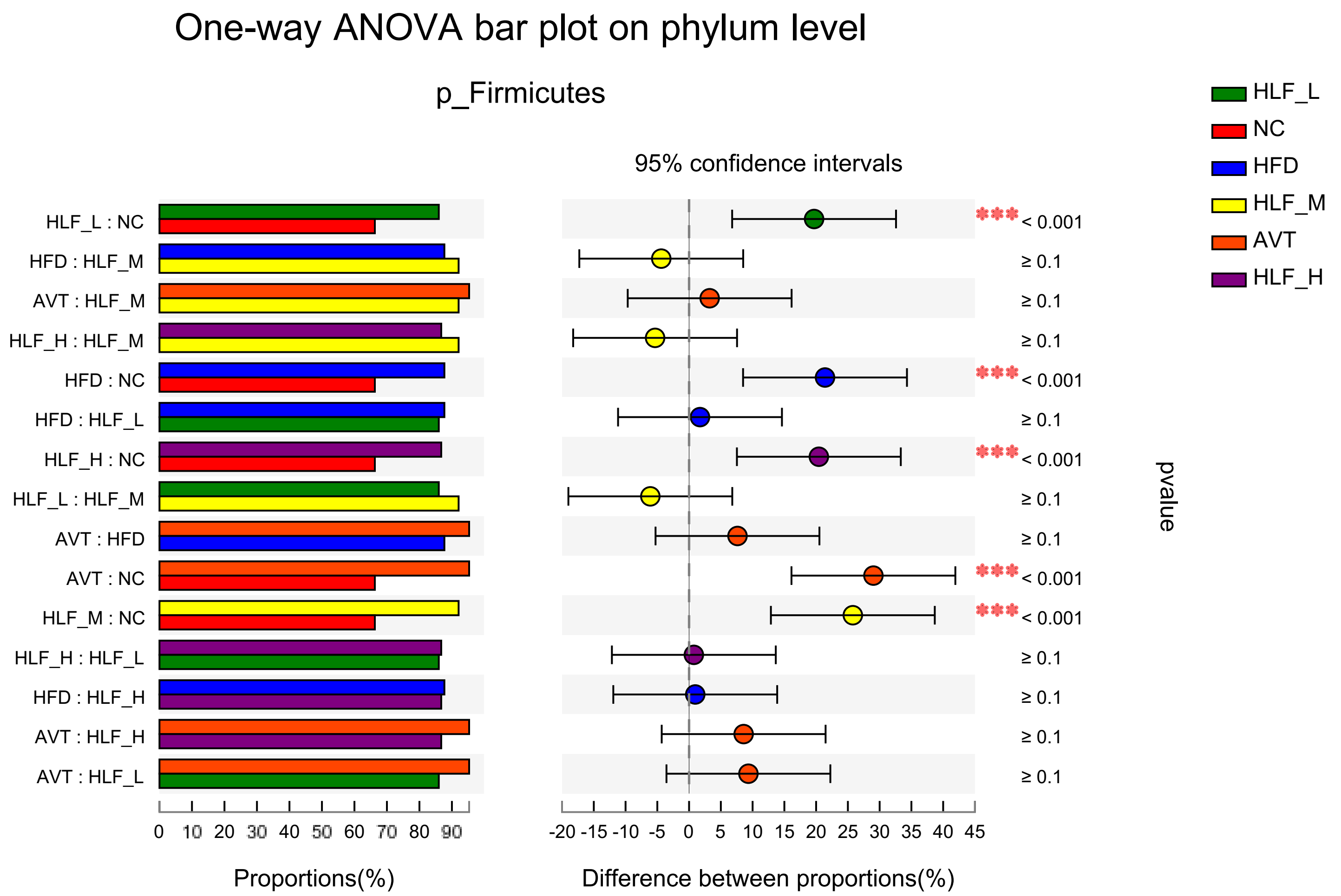
One-way ANOVA bar plot on phylum level
p_Firmicutes
95% confidence intervals
HLF_L : NC
HFD : HLF_M
AVT : HLF_M
HLF_H : HLF_M
HFD : NC
HFD : HLF_L
HLF_H : NC
HLF_L : HLF_M
AVT : HFD
AVT : NC
HLF_M : NC
HLF_H : HLF_L
HFD : HLF_H
AVT : HLF_H
AVT : HLF_L
*** < 0.001
≥ 0.1
≥ 0.1
≥ 0.1
*** < 0.001
≥ 0.1
*** < 0.001
≥ 0.1
≥ 0.1
*** < 0.001
*** < 0.001
≥ 0.1
≥ 0.1
≥ 0.1
≥ 0.1
0 10 20 30 40 50 60 70 80 90
Proportions(%)
-20 -15 -10 -5 0 5 10 15 20 25 30 35 40 45
Difference between proportions(%)
pvalue
HLF_L
NC
HFD
HLF_M
AVT
HLF_H

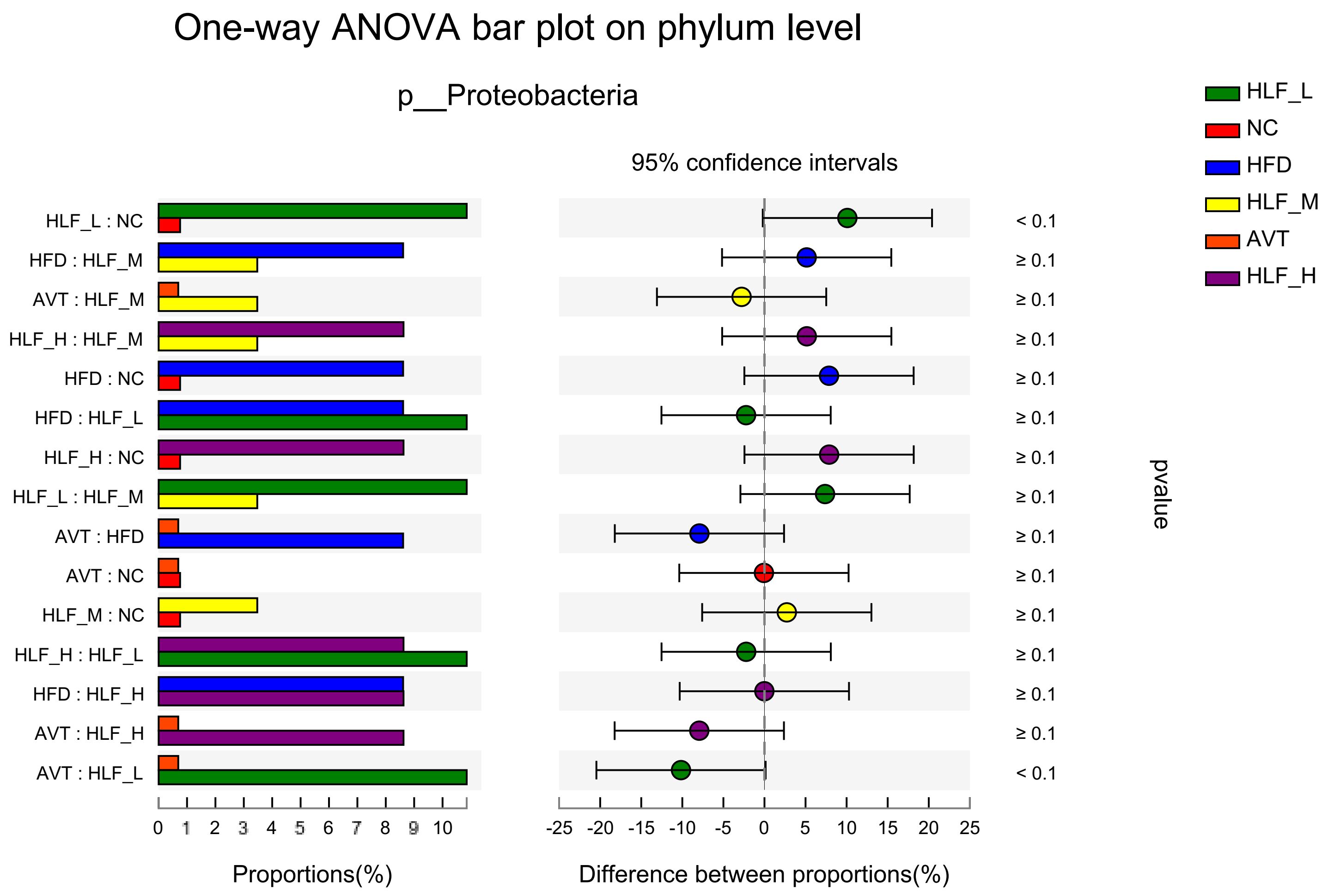

One-way ANOVA bar plot on phylum level
p__Proteobacteria
95% confidence intervals
HLF_L
NC
HFD
HLF_M
AVT
HLF_H
HLF_L : NC
HFD : HLF_M
AVT : HLF_M
HLF_H : HLF_M
HFD : NC
HFD : HLF_L
HLF_H : NC
HLF_L : HLF_M
AVT : HFD
AVT : NC
HLF_M : NC
HLF_H : HLF_L
HFD : HLF_H
AVT : HLF_H
AVT : HLF_L
< 0.1
≥ 0.1
≥ 0.1
≥ 0.1
≥ 0.1
≥ 0.1
≥ 0.1
≥ 0.1
≥ 0.1
≥ 0.1
≥ 0.1
≥ 0.1
≥ 0.1
≥ 0.1
< 0.1
pvalue
0 1 2 3 4 5 6 7 8 9 10
Proportions(%)
-25 -20 -15 -10 -5 0 5 10 15 20 25
Difference between proportions(%)

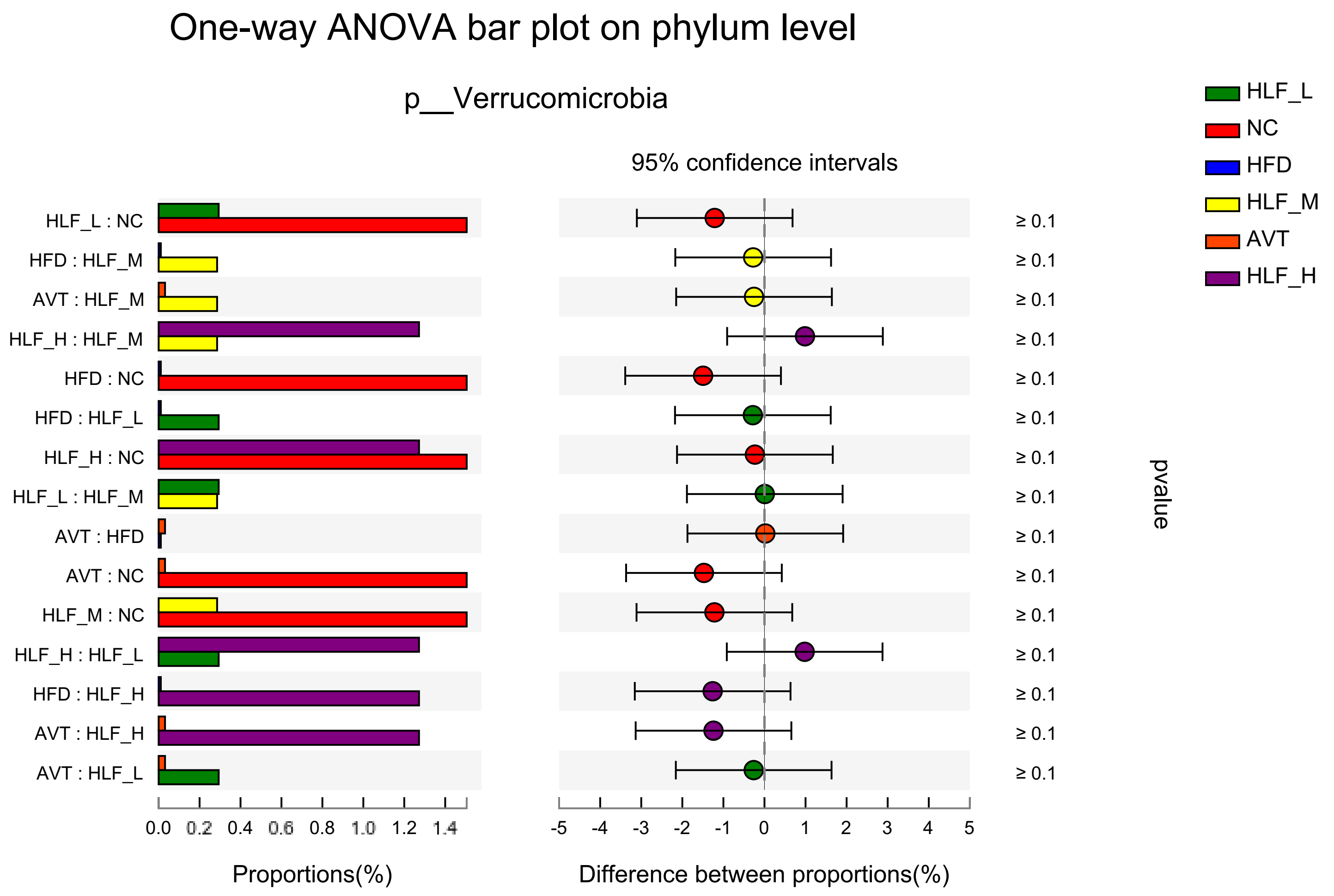
One-way ANOVA bar plot on phylum level
p__Verrucomicrobia
95% confidence intervals
HLF_L
NC
HFD
HLF_M
AVT
HLF_H
HLF_L : NC
HFD : HLF_M
AVT : HLF_M
HLF_H : HLF_M
HFD : NC
HFD : HLF_L
HLF_H : NC
HLF_L : HLF_M
AVT : HFD
AVT : NC
HLF_M : NC
HLF_H : HLF_L
HFD : HLF_H
AVT : HLF_H
AVT : HLF_L
≥ 0.1
pvalue
0.0 0.2 0.4 0.6 0.8 1.0 1.2 1.4
Proportions(%)
-5 -4 -3 -2 -1 0 1 2 3 4 5
Difference between proportions(%)
