# Supplementary material for: The Hypolipidemic Effect of Hawthorn Leaf Flavonoids through Modulating Lipid Metabolism and Gut Microbiota in Hyperlipidemic Rats
Source: Evid Based Complement Alternat Med. 2022 Nov 15;2022:3033311. doi: 10.1155/2022/3033311 (PMC9681556; doi:10.1155/2022/3033311)
Supplement: Supplementary Materials — of this article contain the molecular docking diagram of HLF. Autodock tools-1.5.6 software is used to dock the active ingredients and target proteins of HLF, and the conformation with the lowest docking binding energy is selected for docking mode analysis, and PyMOL software is used for molecular docking mapping and display. Pharmacophore analysis was performed using the Ligplot + program to evaluate hydrogen bonds and van der Waals interaction residues. [file 3033311.f1.zip › Supplementary material 20220708.pdf]

Molecular docking

By analyzing the "active ingredient-target- biological processes" network diagram, we selected the active ingredients of HLF ranked in the top 10 by the degree value, Quercetin, (+)-Catechin, Epicatechin, Rutin, Naringenin, Apigenin, Luteolin, Kaempferol, Myricetin, and Bioquercetin. Based on our previous studies and prediction results, we selected 15 core targets for docking by autodock tools and calculated their binding energies. Plotting the Heatmap according to the binding energy, the greater the absolute value of affinity, the tighter the molecule binds. As molecular docking was not possible due to the zero-charge amount of SREBF2 (PDB: 1UKL), we performed molecular docking visualization between molecules with absolute binding energy greater than 5.37 Å and the protein and plotted the docking diagram. Through the two-dimensional and three-dimensional model maps, we could further observe the spatial structure, binding residues, hydrogen bonding distance, and hydrophobicity between molecules and proteins. As can be seen from the figure, PCSK9, NR1H3 and LDLR showed the most complex molecules bound to the protein molecules (Fig. 1 A-D).

Molecular docking of HLF major compounds with important targets in hyperlipidemia (Fig. 14 B-D). In PCSK9 (PDB ID: 2P4E), the binding free energy of Naringenin to PCSK9 was -5.38 Kcal / mol, in which it formed hydrogen bonds with the amino acid residues of Thr-86, Leu-88, Leu-118, His-116 and Gly-117 with hydrophobic effects (Fig. 1B.a); The binding free energy of Apigenin to PCSK9 was -5.49 Kcal / mol, where it formed hydrogen bonds with the amino acid residues of Leu-118 and Leu-119 with hydrophobic interactions (Fig. 1B.b); Luteolin binds to PCSK9 with a free energy of -5.73 Kcal / mol, where it formed hydrogen bonds with residues Val-114, His-116, Leu-118 and Leu-119, which had hydrophobic effects (Fig. 1B.c); The free energy of binding between Kaempferol and PCSK9 was -6 Kcal/mol, in which it formed hydrogen bonds with the amino acid residues His-113, His-116, Val-114, Leu-118 and Leu-119, and had a hydrophobic effect (Fig. 1B.d).

In NR1H3 (PDB ID: 2ACL), (+)-Catechin has a binding free energy of -5.54 Kcal / mol to NR1H3. In Pymol they formed hydrogen bonds at the amino acid residues of Ser-392, Asp-398, Pro-399 and Phe-402, while in Ligplus they formed hydrogen bonds at the amino acid residues of Val-391, Ser-392, His-395, Asp-398 and Pro-399, and had hydrophobic effects due to the different software performance (Fig. 1C.a); Naringenin showed a binding free energy of -5.57 Kcal / mol to

NR1H3, and in Pymol it formed hydrogen bonds with residues Leu-258, Leu-314, Thr-300 and Arg-303, whereas in Ligplus it formed only hydrogen bonds with residues Met-296 and Thr-300, with hydrophobic interactions (Fig. 1C.b).

In the LDLR (PDB ID: 1LJQ), Naringenin showed a binding free energy of -5.99 Kcal / mol to the LDLR, where it formed hydrogen bonds with the amino acid residues of Ala-416, Gln-639 and Gly-642 residues and had hydrophobic effects (Fig. 1D.a); The binding free energy of Apigenin to the LDLR was -7.27 Kcal / mol, where it formed hydrogen bonds with residues Val-415, Ala-416, Ser-417, Gln-639, Gly-642 and Trp-462 with hydrophobic effects (Fig. 1D.b); The binding free energy of luteolin to LDLR was -5.86 Kcal / mol, in which it formed hydrogen bonds with the amino acid residues of Glu-594, Leu-547, Leu-549 and Leu-637 with hydrophobic interactions (Fig. 1D.c); Kaempferol binds to the LDLR with a free energy of -5.66 Kcal / mol, where it formed hydrogen bonds with residues Leu-547, Leu-549, Val-592, Glu-594 and Thr-638 and had hydrophobic effects (Fig. 1D.d).

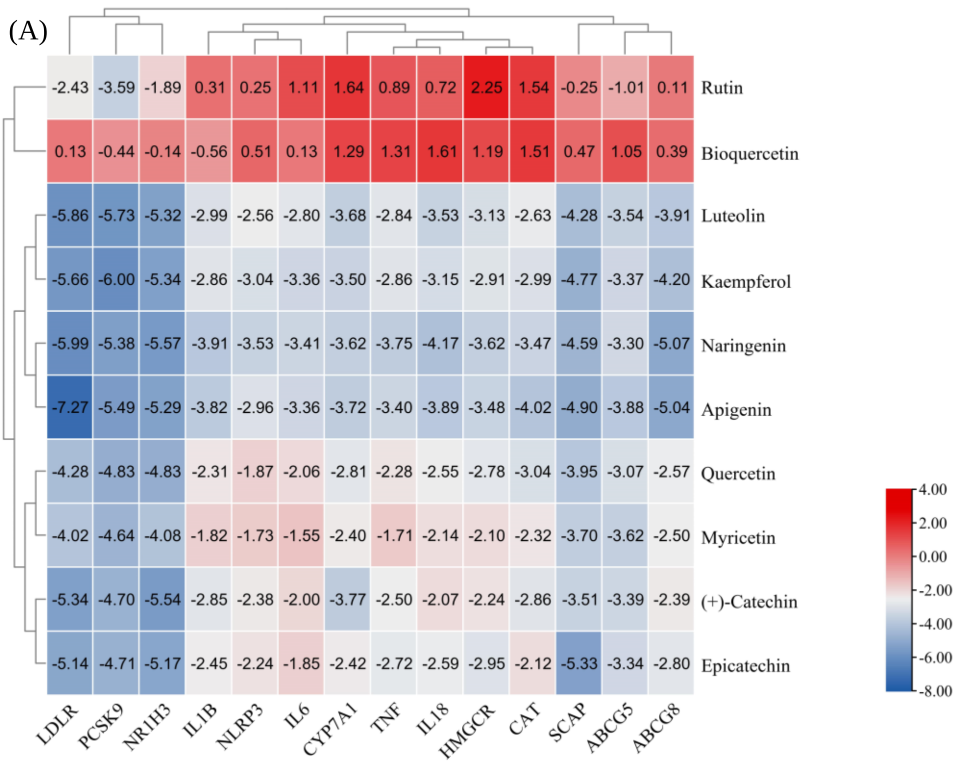

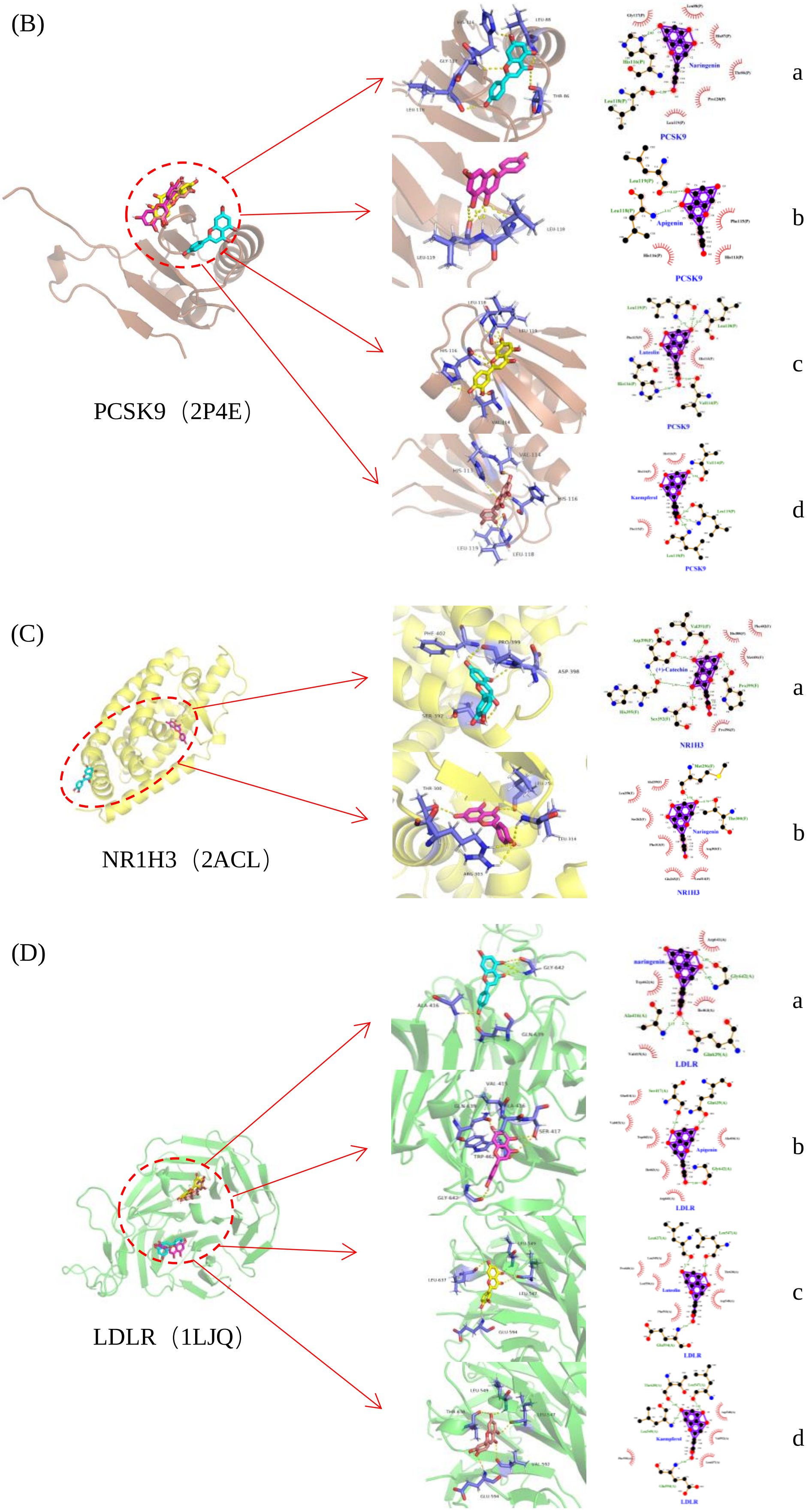

(B)
PCSK9（2P4E）
a
b
c
d
(C)
NR1H3（2ACL）
a
b
(D)
LDLR（1LJQ）
a
b
c
d

Fig.1. Molecular docking diagram of HLF. (A) Heatmap of HLF docking energy; (B) Compound molecule interacts with PCSK9; (C) Compound molecule interacts with NR1H3; (D) Compound molecule interacts with LDLR.
